# Perioperative Schmerztherapie mit Nichtopioidanalgetika: Gemeinsame interdisziplinäre Empfehlung der Deutschen Schmerzgesellschaft, der Deutschen Gesellschaft für Anästhesiologie und Intensivmedizin und der Deutschen Gesellschaft für Chirurgie

**DOI:** 10.1007/s00104-021-01421-w

**Published:** 2021-05-26

**Authors:** Ulrike M. Stamer, Joachim Erlenwein, Stephan M. Freys, Thomas Stammschulte, Dirk Stichtenoth, Stefan Wirz

**Affiliations:** 1 Universitätsklinik für Anästhesiologie und Schmerztherapie, Inselspital, Universität Bern, Freiburgstrasse, 3010 Bern, Schweiz; 2 Arbeitskreis Akutschmerz, Deutsche Schmerzgesellschaft e.V., Berlin, Deutschland; 3 Klinik für Anästhesiologie, Universitätsmedizin Göttingen, Göttingen, Deutschland; 4 Wissenschaftlicher Arbeitskreis Schmerzmedizin, Deutsche Gesellschaft für Anästhesiologie und Intensivmedizin e.V., Nürnberg, Deutschland; 5 Chirurgische Klinik, DIAKO Ev. Diakonie-Krankenhaus Bremen, Bremen, Deutschland; 6 Chirurgische Arbeitsgemeinschaft Akutschmerz, Deutsche Gesellschaft für Chirurgie e.V., Berlin, Deutschland; 7 Bern, Schweiz; 8 ehemalige Institution Arzneimittelkommission der deutschen Ärzteschaft, Berlin, Deutschland; 9 Institut für Klinische Pharmakologie, Medizinische Hochschule Hannover, Hannover, Deutschland; 10 Abteilung für Anästhesie, Interdisziplinäre Intensivmedizin, Schmerzmedizin/Palliativmedizin, Zentrum für Schmerzmedizin, Weaningzentrum, CURA – GFO-Kliniken Bonn, Bad Honnef, Deutschland; 11 Arbeitskreis Tumorschmerz, Deutsche Schmerzgesellschaft e.V., Berlin, Deutschland

**Keywords:** Akutschmerztherapie, Metamizol, Nichtopioidanalgetika, NSAIDs, Paracetamol, Patienteninformation, Perioperativ, Acute pain, Metamizole (Dipyrone), Nonopioid analgesics, NSAIDs, Paracetamol, Perioperative pain management, Patient information

## Abstract

**Hintergrund:**

Nichtopioidanalgetika werden bei vielen Patienten zur perioperativen Analgesie eingesetzt. Zu einigen praktischen Fragen beim Einsatz von Nichtopioidanalgetika liegen z. T. nur wenig Informationen aus Studien vor, und in Krankenhäusern existieren häufig keine Konzepte zum Vorgehen, z. B. zur Patientenaufklärung und zum Zeitpunkt der perioperativen Gabe.

**Methodik:**

Eine Expertengruppe der beteiligten Fachgesellschaften hat konsensbasierte Empfehlungen zum perioperativen Einsatz von Nichtopioidanalgetika erarbeitet und in einem strukturierten formalen Konsensusprozess verabschiedet.

**Ergebnisse:**

Die Arbeitsgruppe stimmt überein, dass Nichtopioidanalgetika Bestandteil eines perioperativen multimodalen Analgesiekonzepts sein sollen und Patienten präoperativ über Nutzen, Risiken und alternative Behandlungsmöglichkeiten aufgeklärt werden sollen. Die präoperative Patienteninformation und -edukation soll auch eine Schmerz- und Analgetikaanamnese umfassen und Patienten mit Risikofaktoren für starke Schmerzen und eine Schmerzchronifizierung sollen identifiziert werden. Unter Berücksichtigung von Kontraindikationen können Nichtopioidanalgetika abhängig von der Operationsdauer auch schon prä- oder intraoperativ gegeben werden, um nach Beendigung der Anästhesie ausreichende Plasmakonzentrationen zu erzielen. Nichtopioidanalgetika oder Kombinationen von (Nichtopioid‑)Analgetika sollen nur für einen begrenzten Zeitraum gegeben werden. Ein gemeinsam erarbeiteter abteilungsübergreifender Behandlungsstandard mit dem Nichtopioidanalgetikum erster Wahl, weiteren Therapieoptionen sowie adäquaten Dosierungen, ergänzt durch eingriffsspezifische Konzepte, soll schriftlich hinterlegt werden. Bei Entlassung aus dem Krankenhaus soll der nachbehandelnde Arzt zu perioperativ gegebenen und aktuell noch eingenommenen Analgetika schriftliche Informationen erhalten. Patienten sollen zu möglichen Nebenwirkungen der Analgetika und ihrer Symptome, die auch nach Krankenhausentlassung auftreten können, und die befristete Einnahmedauer informiert werden.

**Schlussfolgerung:**

Die Anwendung von Nichtopioidanalgetika soll als Bestandteil eines perioperativen multimodalen Analgesiekonzepts mit klaren Vorgaben zu Indikationen, Kontraindikationen, Dosierungen und Behandlungsdauer in einem abteilungsübergreifenden Behandlungsstandard schriftlich hinterlegt werden.

**Zusatzmaterial online:**

Die Offenlegung von Interessen ist in der Online-Version dieses Artikels (10.1007/s00104-021-01421-w) enthalten.

## Abstract (en)

**Background:**

Nonopioid analgesics are frequently used for perioperative analgesia; however, insufficient research is available on several practical issues. Often hospitals have no strategy for how to proceed, e.g., for informing patients or for the timing of perioperative administration of nonopioid analgesics.

**Methods:**

An expert panel representing the German national societies of pain, anaesthesiology and intensive care medicine and surgery developed recommendations for the perioperative use of nonopioid analgesics within a formal, structured consensus process.

**Results:**

The panel agreed that nonopioid analgesics shall be part of a multimodal analgesia concept and that patients have to be informed preoperatively about possible complications and alternative treatment options. Patients’ history of pain and analgesic intake shall be evaluated. Patients at risk of severe postoperative pain and possible chronification of postsurgical pain shall be identified. Depending on the duration of surgery, nonopioid analgesics can already be administered preoperatively or intraoperatively so that plasma concentrations are sufficient after emergence from anesthesia. Nonopioid analgesics or combinations of analgesics shall be administered for a limited time only. An interdisciplinary written standard of care, comprising the nonopioid analgesic of choice, possible alternatives, adequate dosing and timing of administration as well as surgery-specific policies, have to be agreed upon by all departments involved. At discharge, the patient’s physician shall be informed of analgesics given and those necessary after discharge. Patients shall be informed of possible side effects and symptoms and timely discontinuation of analgesic drugs.

**Conclusion:**

The use of nonopioid analgesics as part of a perioperative multimodal concept should be approved and established as an interdisciplinary and interprofessional concept for the adequate treatment of postoperative pain.

Die perioperative Gabe von Nichtopioidanalgetika (NOPA) bei Erwachsenen ist etabliert und wird im Kontext analgetischer Konzepte von verschiedenen nationalen und internationalen Leitlinien empfohlen [18, 79].

Zu den NOPA zählen Paracetamol, Metamizol und die NSAIDs („nonsteroidal anti-inflammatory drugs“), welche in nichtselektive Cyclooxygenase(COX)-Inhibitoren (traditionelle NSAIDs) und selektive COX-2-Inhibitoren (Coxibe) unterteilt werden. In der perioperativen Phase dienen NOPA meist als Basis einer systemischen Analgesie und können mit Opioiden und ggf. Koanalgetika in ein multimodales Analgesiekonzept integriert werden. Zusätzlich benötigte Opioide sollten in der frühen postoperativen Phase bedarfsabhängig titriert werden und können im weiteren Verlauf als orale Medikation fortgeführt werden. Durch Kombination von NOPA und Opioiden können der Opioidbedarf und zum Teil auch opioidtypische Nebenwirkungen, wie Übelkeit, Erbrechen, Müdigkeit und Sedierung, reduziert werden [27, 48, 49]. Für NOPA wurden in den letzten Jahren zunehmend schwerwiegende oder gar tödliche Nebenwirkungen diskutiert. Hierzu zählen z. B. kardiovaskuläre Ereignisse, die zunächst für Coxibe, später auch für traditionelle NSAIDs beschrieben wurden, oder die Agranulozytose unter Metamizol. Zu einigen konkreten Fragen der perioperativen Anwendung von NOPA bei Erwachsenen liegt jedoch keine oder wenig Evidenz aus klinischen Studien vor. Dabei geht es häufig um praktische Fragen in der täglichen klinischen Anwendung, z. B. um die Patientenaufklärung oder den Zeitpunkt der perioperativen Gabe. Oftmals liegen nur wenige Informationen aus Studien vor und in Krankenhäusern existieren häufig keine Konzepte zum Vorgehen.

Die Empfehlungen umfassen keine detaillierte Nutzen-Risiko-Analysen von NOPA im Vergleich zu alternativen Maßnahmen in der perioperativen Akutschmerztherapie wie Opioide, Koanalgetika, Verfahren der Regional- bzw. Lokalanästhesie und psychologische Interventionen. Hierfür verweisen wir auf die bald erscheinenden überarbeiteten S3-Leitlinien „Behandlung akuter perioperativer und posttraumatischer Schmerzen“. Die S3-Leitlinien können möglicherweise einige der hier ausgesprochenen Empfehlungen in ihrem Empfehlungsgrad geringfügig modifizieren.

## Methodik

Die Expertengruppe setzte sich zusammen aus Vertretern des Arbeitskreises Akutschmerztherapie der Deutschen Schmerzgesellschaft, des wissenschaftlichen Arbeitskreises Schmerzmedizin der Deutschen Gesellschaft für Anästhesiologie und Intensivmedizin (DGAI) und der Chirurgischen Arbeitsgemeinschaft Akutschmerz (CAAS) der Deutschen Gesellschaft für Chirurgie (DGCH). Ebenso beteiligt waren Vertreter der Arzneimittelkommission der deutschen Ärzteschaft (AkdÄ) sowie ein klinischer Pharmakologe. Jeder beteiligte Experte legte seine potenziellen Interessenkonflikte anhand des entsprechenden AWMF-Dokuments offen.

Neben einer ausführlichen Diskussion der neueren Befunde zu kardiovaskulären Nebenwirkungen, die als Hintergrundinformation ebenfalls dargestellt werden, wurden folgende Aspekte zum perioperativen Einsatz von NOPA thematisiert:

I. Allgemeine Empfehlungen
  Indikation für NOPA
  NOPA und kardiovaskuläre Vorerkrankungen
  Kombination von NOPA
II. Präoperative Evaluation und Planung der Schmerztherapie
  Anamnese
  Risikopatienten identifizieren
  Präoperative Planung der perioperativen Analgesie
  Präoperative Patienteninformation zu NOPA
III. NOPA prä- und intraoperativ
  Zeitpunkt der prä- und intraoperativen Gabe von NOPA
  Intraoperative Hypovolämie und Nierenfunktion
IV. NOPA postoperativ
  Anwendungsdauer der NOPA postoperativ
  Abteilungsübergreifender Behandlungsstandard mit NOPA
  Information an den nachbehandelnden Arzt
  Informationspflicht des Arztes

Die Empfehlungen zur präoperativen Evaluation, Patienteninformation und Beteiligung sowie die Thematisierung abteilungsübergreifender Konzepte berücksichtigen auch den Beschluss des Gemeinamen Bundesausschusses (unabhängiges Gremium aus Vertretern der gesetzlichen Krankenkassen, der Deutschen Krankenhausgesellschaft, der kassenärztliche Bundesvereinigungen, der Patientenvertretung) zu einrichtungsinternen Konzepten der Akutschmerztherapie mit entsprechenden Qualitätsmanagementrichtlinien [14].

Im Oktober 2018 wurden Kernaussagen und ihre Formulierungen zur perioperativen Anwendung von NOPA in einem Arbeitskreis diskutiert und eine erste Version erstellt. Die Empfehlungsgraduierung richtete sich nach den Vorgaben der Arbeitsgemeinschaft der Wissenschaftlichen Medizinischen Fachgesellschaften (AWMF) mit einer Graduierung der Empfehlung: starke Empfehlung „soll“, Empfehlung „sollte“ oder offene Empfehlung „kann“ und entsprechende Verneinung für die drei Empfehlungsgrade [5].

Eine formale Konsentierung erfolgte im Rahmen eines Delphi-Verfahrens. Dafür erhielten die Beteiligten per E‑Mail den Link für eine anonyme Onlineabstimmung (SurveyMonkey, Inc., Palo Alto, California, USA, 2016) und parallel den Gesamttext der Empfehlungen. Entsprechend den Rückmeldungen wurden Formulierungen überarbeitet und erneut zur Kommentierung versandt mit einem abschließenden Votum nach dem externen Reviewverfahren Anfang 2021. Jede Empfehlung sollte eine mindestens 75 %ige Zustimmung in der Expertengruppe finden. Die Konsensstärke für jede Empfehlung wird im Anhang dargestellt.

## Hintergrund

NOPA werden perioperativ bei vielen Patienten eingesetzt und gehören zu den Standardanalgetika in der Akutschmerztherapie. Da vor allem die kardiovaskulären Nebenwirkungen in den letzten Jahren vielfältig untersucht worden sind, soll im Folgenden eine kurze Zusammenfassung dieser Ergebnisse aus der Perspektive der Perioperativmedizin als Hintergrundinformation erfolgen.

## Nebenwirkungen und kardiovaskuläres Risiko der NOPA

Nebenwirkungen können bei allen NOPA auftreten (Übersichten zu Wirkmechanismen, Nebenwirkungen und Prophylaxe von Nebenwirkungen z. B. in [37, 77, 79]). Insgesamt sind schwere Komplikationen sehr selten, vor allem bei kurzzeitigem Einsatz und Beachtung der Kontraindikationen und Warnhinweise.

### Paracetamol

Paracetamol wurde über Jahrzehnte als „harmlos“ angesehen. Nach der zunächst in experimentellen Untersuchungen gezeigten COX-2-Hemmung [39, 41] wurden auch in klinischen Studien vermehrt kardiovaskuläre Ereignisse (Erhöhung des Blutdrucks, Herzinfarkt, Schlaganfall) vor allem bei Risikopatienten beschrieben [13, 17, 93, 94, 96]. Jedoch gibt es auch Studien, die dieses nicht bestätigt haben [40, 41, 77]. Ein Review aus dem Jahr 2018 belegt für die Langzeittherapie mit Paracetamol ein erhöhtes, dosisabhängiges Risiko für gastrointestinale Blutungen sowie einen Blutdruckanstieg von ~4 mm Hg [50]. Entsprechende Untersuchungen für den perioperativen bzw. kurzzeitigen Einsatz liegen nicht vor.

Paracetamol hat eine geringe therapeutische Breite mit dosisabhängiger Lebertoxizität. Die vorgegebene maximale Tagesdosis von 4 g für Patienten >50 kg und ohne Komorbidität soll nicht überschritten werden. Eine mögliche Eigenmedikation mit freiverkäuflichem Paracetamol und Kombinationspräparaten, wie z. B. Erkältungsmedikamente, sollte berücksichtigt werden. CAVE: Bei Leberzirrhose Child-Pugh A oder B und zusätzlichen Risikofaktoren für Hepatotoxizität (Mangelernährung oder Alkoholkonsum) sowie Leberzirrhose Child-Pugh C soll die Dosis auf maximal 2 g/Tag reduziert werden [90].

### Metamizol

Metamizol ist seit 1922 auf dem Markt und wird ebenfalls nicht zu den klassischen NSAIDs gezählt. Es hat keine periphere antiphlogistische Wirkung, obwohl der aktive Metabolit 4‑N-Methylaminoantipyrin (4-MAA) unselektiv die COX inhibiert [40, 41, 77]. Der Wirkmechanismus von Metamizol weicht von dem der NSAIDs ab. Metamizol bildet einen stabilen Komplex mit dem Häm, wodurch Radikale abgefangen werden, die die katalytische Aktivität des Enzyms induzieren [68]. Zahlreiche weitere durch Metamizol vermittelte periphere und zentrale antinozizeptive Effekte sind beschrieben worden [40, 68, 70]. Im Vergleich zu den NSAIDs fehlt Metamizol die antiphlogistische Wirkung sowie die typischen Nebenwirkungen der COX-Inhibitoren [37]. Metamizol ist für den Gastrointestinaltrakt besser verträglich als NSAIDs und hat im Vergleich zu Paracetamol und NSAIDs eine große therapeutische Breite [4, 37, 40, 81]. Für eine Nephrotoxizität durch perioperativ verabreichtes Metamizol liegt gegenwärtig keine hinreichende Evidenz vor [37, 88].

Anaphylaktische bzw. anaphylaktoide Reaktionen sind mit einer Häufigkeit von 1:5000 beschrieben. Gefährdet sind vor allem Patienten mit Analgetikaasthma, Analgetikaintoleranz vom Urtikariaangioödemtyp, Asthma bronchiale, chronischer Urtikaria, Unverträglichkeit von Alkohol, Farb- und Konservierungsstoffen (z. B. Benzoate). Die EMA (European Medicines Agency) weist 2020 erstmalig auch auf einen arzneimittelbedingten Leberschaden im Zusammenhang mit einer Metamizoleinnahme hin [28]. Die genaue Häufigkeit kann nicht berechnet werden, wird jedoch als sehr selten eingeschätzt [28].

Vor allem bei (zügiger) i.v. Applikation kann es zu isolierten, möglicherweise dosisabhängigen, kritischen Hypotensionen kommen. Gefährdet sind vor allem Patienten mit Volumenmangel, Dehydratation, instabilem Kreislauf/beginnendem Kreislaufversagen oder hohem Fieber. Für die i.v. Gabe ist eine Kurzinfusion über ca. 20–30 min mit Kreislaufüberwachung empfehlenswert. Wird der Patient symptomatisch, sollte die Infusion gestoppt werden. Für Details zur metamizolinduzierten Agranulozytose verweisen wir auf die 2019 publizierten Empfehlungen [87].

Metamizol wurde bislang nicht mit kardiovaskulären Nebenwirkungen in Verbindung gebracht. In einer Studie zu NOPA und kardiovaskulären Ereignissen ergab sich für Patienten mit niedrigem, mittlerem oder hohem kardiovaskulärem Risiko unter Langzeittherapie mit Metamizol kein erhöhtes Risiko für einen Myokardinfarkt [23].

### Traditionelle NSAIDs und Coxibe

Wirkmechanismus und Risikoprofil von traditionellen NSAIDs und Coxiben sind in den letzten Jahren intensiv untersucht worden (Übersichten [37, 77, 79]). Der analgetische Effekt beruht auf einer Hemmung der COX‑2, während für Nebenwirkungen sowohl die COX-1- als auch die COX-2-Hemmung eine Rolle spielen. Es soll an dieser Stelle nicht auf alle in den Fachinformationen beschriebenen Nebenwirkungen der einzelnen Substanzen eingegangen werden, wie z. B. gastrointestinale Nebenwirkungen (Ulzera, Blutungen, Perforationen), Nieren‑, Leberschäden, Einfluss auf die Blutgerinnung, Allergien und Asthmaanfälle. Seit der Marktrücknahme von Rofecoxib 2004 wurden jedoch kardiovaskuläre Nebenwirkungen von Coxiben und traditionellen NSAIDs intensiv untersucht und diese Befunde haben auch Implikationen für die perioperative Anwendung. Die Studienergebnisse führten zu einer deutlichen Einschränkung der Anwendung von traditionellen NSAIDs und Coxiben. Zusätzliche Kontraindikationen wurden in die Fachinformationen aufgenommen, allerdings bis jetzt mit einer fehlenden Differenzierung zwischen Lang- und Kurzzeitgabe. Im nächsten Abschnitt wird der aktuelle Stand hinsichtlich COX-Hemmung und kardiovaskulärer Ereignisse zusammengefasst.

### Kardiovaskuläres Risiko

Durch Hemmung der endothelialen COX‑2 wird die Synthese von Prostacyclin, das antiaggregatorisch und vasodilatativ wirkt, inhibiert. Einen gegenläufigen Effekt vermittelt eine COX-1-Hemmung in den Thrombozyten. Die Synthese des proaggregatorischen und vasokonstriktiven Thromboxan A2 ist COX-1-abhängig, wird also vor allem durch traditionelle NSAIDs, weniger durch Coxibe unterbunden. Durch das Überwiegen der COX-2-Hemmung, also mehr prothrombotischer als antithrombotischer Effekte, werden kardio- bzw. zerebrovaskuläre Ereignisse begünstigt. Hinzu kommen weitere Effekte wie Flüssigkeitsretention und Blutdruckanstieg. Dadurch können traditionelle NSAIDs und Coxibe zu einer Erhöhung des Risikos für thromboembolische vaskuläre Ereignisse und Herzinsuffizienz führen. Das Ausmaß des Risikos variiert je nach Substanz, deren jeweiliger Potenz, die COX‑1 und COX‑2 zu hemmen, und der Dosis [37, 77]. Die Gegenanzeigen und Warnhinweise in den Fach- und Gebrauchsinformationen sind daher für die Wirkstoffe unterschiedlich (Beispiele in Tab. 1). Während für Ibuprofen und Naproxen (präferenzielle COX-1-Inhibition) explizit nur die schwere Herzinsuffizienz als Gegenanzeige genannt wird, kommen für Diclofenac, Parecoxib und Celecoxib (präferenzielle bzw. selektive COX-2-Inhibition) weitere hinzu (Tab. 1).

**Table 1:** 

Medikament	Gegenanzeige	Warnhinweise/relative Kontraindikationen → sorgfältige Nutzen-Risiko-Abwägung bei:
Celecoxib	Herzinsuffizienz (NYHA II–IV)	Patienten mit erheblichen Risikofaktoren für das Auftreten kardiovaskulärer Ereignisse (z. B. Bluthochdruck, Hyperlipidämie, Diabetes mellitus, Rauchen)
	Klinisch gesicherte KHK	
	Periphere arterielle Verschlusskrankheit	
	Zerebrovaskuläre Erkrankungen	
Diclofenac	Bekannte Herzinsuffizienz (NYHA II–IV)	Unkontrolliertem Bluthochdruck
	Ischämische Herzkrankheit	Kongestiver Herzinsuffizienz NYHA I
	Periphere arterielle Verschlusskrankheit	Patienten mit signifikanten Risikofaktoren für kardiovaskuläre Ereignisse (z. B. Hypertonie, Hyperlipidämie, Diabetes mellitus, Rauchen)
	Zerebrovaskuläre Erkrankung	
Ibuprofen	Schwere Herzinsuffizienz	Unkontrolliertem Bluthochdruck
		Herzinsuffizienz
		Bestehender ischämischer Herzerkrankung
		Peripherer arterieller Verschlusskrankheit
		Zerebrovaskulärer Erkrankung
		Patienten mit Risikofaktoren für kardiovaskuläre Ereignisse (z. B. Bluthochdruck, Hyperlipidämie, Diabetes mellitus, Rauchen) vor Initiierung einer länger dauernden Behandlung
Naproxen	Schwere Herzinsuffizienz	Unkontrolliertem Bluthochdruck
		Herzinsuffizienz
		Bestehender ischämischer Herzerkrankung
		Peripherer arterieller Verschlusskrankheit
		Zerebrovaskulärer Erkrankung
		Patienten mit Risikofaktoren für kardiovaskuläre Ereignisse (z. B. Bluthochdruck, Hyperlipidämie, Diabetes mellitus, Rauchen) vor Initiierung einer länger dauernden Behandlung
Parecoxib	Herzinsuffizienz (NYHA II–IV)	Patienten mit erheblichen Risikofaktoren für das Auftreten kardiovaskulärer Ereignisse (z. B. Bluthochdruck, Hyperlipidämie, Diabetes mellitus, Rauchen)
	Behandlung postoperativer Schmerzen nach einer koronaren Bypassoperation	
	Klinisch gesicherte KHK	
	Periphere arterielle Verschlusskrankheit	
	Zerebrovaskuläre Erkrankungen	

#### Wichtig für die klinische Praxis.

Wird die Dosis der NSAIDs reduziert, um durch eine weniger ausgeprägte COX-2-Inhibition kardiovaskuläre Ereignisse zu reduzieren, so wird auch die über COX‑2 vermittelte analgetische Wirkung vermindert [37].

Die meisten Studien zur kardiovaskulären Sicherheit von traditionellen NSAIDs und Coxiben beziehen sich auf die Langzeittherapie, vor allem von Arthrose und Arthritis [10, 59, 83]. Naproxen schneidet hinsichtlich des kardiovaskulären Risikos meist am günstigsten ab, gefolgt von niedrig dosiertem Ibuprofen und Celecoxib. Ein höheres Risiko wurde in einer Studie für Diclofenac und Etoricoxib errechnet [52, 52], in einer anderen für Ibuprofen und Diclofenac [83]. In der PRECISION-Studie wurde Celecoxib 200 mg/Tag gegen Naproxen (850 mg) und Ibuprofen (2000 mg) über 20 Wochen mit fast 3 Jahren Nachbeobachtungszeit verglichen [59]. Celecoxib zeigte bei Patienten mit vorbestehenden kardiovaskulären Risikofaktoren hinsichtlich kardiovaskulärer Ereignisse keine schlechteren Ergebnisse als die beiden traditionellen NSAIDs, hinsichtlich renaler und gastrointestinaler Ereignisse (Ulkus, Blutung, Perforation) war es besser oder gleich gut wie Ibuprofen bzw. Naproxen [10, 37]. Hingegen wurden unter Naproxen (1000 mg/Tag) mit seiner ausgeprägte Affinität zur COX‑1 vermehrt Blutungen im oberen Gastrointestinaltrakt nachgewiesen [10, 37]. Bei paralleler Gabe von Antikoagulanzien wird das Blutungsrisiko zusätzlich erhöht [37]. Eine ergänzende Medikation mit Protonenpumpeninhibitoren wird daher empfohlen. Insgesamt wird Naproxen auch wegen seiner sehr langen Halbwertszeit von 12–15 h [37] perioperativ kaum genutzt.

Für Ibuprofen bestätigt die EMA nach einer umfassenden Bewertung ein leicht erhöhtes Risiko für kardiovaskuläre Ereignisse bei Langzeitanwendung von hohen Dosen (≥2400 mg). Sie empfiehlt, bei Patienten mit kardiovaskulären Vorerkrankungen (schlecht eingestellte arterielle Hypertonie, Herzinsuffizienz, KHK, periphere arterielle Verschlusskrankheit, zerebrovaskuläre Erkrankung) keine hohen Dosen zu verwenden. Damit wird das Risiko von hoch dosiertem Ibuprofen vergleichbar eingestuft wie das von Diclofenac oder Rofecoxib. Hingegen sah die EMA bei niedrigeren Dosierungen von bis zu 1200 mg Ibuprofen/Tag kein erhöhtes Risiko [10, 61, 62, 72].

Relevant für die perioperative Analgesie sind Analysen des dänischen Patientenregisters: Das kardiovaskuläre Risiko durch NSAIDs als Gesamtgruppe war bei Patienten mit vorausgegangenem Myokardinfarkt auch schon nach 7 Tagen Behandlung erhöht, für Diclofenac isoliert betrachtet sogar schon direkt ab Behandlungsbeginn. Die Autoren schließen daraus, dass es kein sicheres Behandlungsfenster für NSAIDs gebe [73, 74]. Die weitere Datenauswertung ergab, dass für mindestens 5 Jahre nach einem Myokardinfarkt das kardiovaskuläre Risiko durch NSAID-Einnahme im Vergleich zu Patienten ohne NSAID-Therapie erhöht blieb [62]. Kanadische Untersuchungen von Versicherungsdaten und eingelösten Rezepten bestätigen ein erhöhtes Infarktrisiko schon innerhalb der ersten Behandlungswoche mit einem NSAID. Dieses Risiko war im ersten Behandlungsmonat unter hohen Dosen am höchsten [9]. Obwohl die Datenlage zur perioperativen Kurzzeitgabe von NSAIDs momentan unzureichend ist, sollten die Kontraindikationen der Fachinformationen auch für die Akutschmerztherapie beachtet werden.

### Interaktionen von NOPA mit ASS (Acetylsalicylsäure)

In-vitro-Untersuchungen von Plasmaproben zeigten, dass Metamizol die durch ASS (Acetylsalicylsäure: 75–150 mg/Tag) induzierte Inhibition der Thrombozytenaggregation aufheben kann [75]. Dieses wurde in Pilotstudien an kleinen Patientenkollektiven mit kardiovaskulären Risikofaktoren bzw. nach einem Apoplex teilweise bestätigt, wenn ASS und Metamizol zeitgleich eingenommen wurden [1, 21, 66]. In der Metamizol-Fachinformation wurde daher folgender Text ergänzt: „Metamizol kann bei gleichzeitiger Anwendung die Wirkung von Acetylsalicylsäure auf die Thrombozytenaggregation vermindern. Daher sollte Metamizol bei Patienten, die Acetylsalicylsäure in niedriger Dosierung zur Kardioprotektion einnehmen, mit Vorsicht angewendet werden“. Nach Beschluss der EMA soll dieser Hinweis auch bei allen ASS-Präparaten zur Thrombozytenaggregation in die Fachinformation aufgenommen werden [30].

Daten zur klinischen Relevanz aus Untersuchungen mit hohen Patientenzahlen fehlen bisher. Die Arzneimittelkommission der deutschen Ärzteschaft kommentierte hierzu, dass noch keine abschließende Beurteilung möglich sei [6]. In einem Übersichtsartikel, der die Literatur bis Anfang 2019 einschließt, wird ebenfalls auf die noch nicht eindeutige Datenlage bei fehlenden Replikationsstudien hingewiesen [76]. Vermutlich spielt bei kurzfristiger Einnahme von Metamizol im Rahmen der perioperativen Schmerztherapie diese Interaktion nur eine untergeordnete Rolle [21, 76, 99]. Bei einer strikten Einhaltung der Einnahmereihenfolge, zuerst ASS, und (mindestens) 30 min später Metamizol, bleibt der ASS-Effekt auf die Thrombozytenaggregation hingegen erhalten [22]. Weiterhin haben auch Dosis und Applikationsweg (Metamizol oral unproblematischer als intravenös) einen Einfluss auf diese Medikamenteninteraktion. Zumindest während des stationären Aufenthalts eines Patienten ließe sich die zeitversetze Gabe der beiden Substanzen umsetzen. Da diese Interaktion intensiv erforscht wird, werden in nächster Zeit eventuell neue Daten vorliegen, die spezifischere Empfehlungen erlauben.

Für die Kombination von Ibuprofen und niedrig dosiertem ASS wird schon seit längerem eine Abschwächung der ASS-Wirkung diskutiert [16], die bei kurzfristigem Einsatz von Ibuprofen wahrscheinlich wenig relevant ist. Die EMA hat 2015 folgenden Hinweis gegeben [29]:

Laboratory studies have shown that ibuprofen reduces the blood-thinning effects of aspirin. However, it remains uncertain whether long-term use of ibuprofen in clinical practice reduces the benefits of low-dose aspirin in preventing heart attacks and strokes. Occasional use of ibuprofen should not affect the benefits of low-dose aspirin.

Um dieses Problem zu umgehen, wurde eine zeitlich versetze Einnahme oder der Einsatz eines alternativen NSAID empfohlen [2, 32]. Interaktionen mit ASS sind auch für weitere NSAIDs beschrieben [32]. Für Naproxen wird diese Interaktion in der Fachinformation ebenfalls mit dem Zusatz adressiert, dass die klinische Relevanz nicht bekannt sei. Ein systemisches Review von 32 Studien einer kanadischen Autorengruppe attestiert eine Diskrepanz zwischen In-vitro‑, In-vivo- und Ex-vivo-Untersuchungen hinsichtlich der durch Ibuprofen, Naproxen und Celecoxib induzierten Reduktion des ASS-Effekts auf die Thrombozytenaggregation [2]. Ein klinisch ungünstigeres kardiovaskuläres Outcome von Patienten unter dieser Komedikation konnten die Autoren nicht bestätigen. Ähnliche Ergebnisse wurden von anderen Arbeitsgruppen berichtet [23, 65].

Grundsätzlich sollte jedoch die perioperative Gabe jeglicher NSAIDs bei bestehender niedrig dosierter ASS-Einnahme hinterfragt werden. Nimmt ein Patient ASS, ist eine kardiovaskuläre Komorbidität naheliegend, die dann eine Kontraindikation für NSAIDs darstellt (Tab. 1).

## I. Allgemeine Empfehlungen

### 1. Indikation für NOPA

NOPA gelten als wichtiger Bestandteil eines perioperativen multimodalen Analgesieregimes. Allerdings sind postoperative Schmerzen nicht explizit in allen Fachinformationen der entsprechenden Präparate als Indikation aufgeführt (Tab. 2). Häufig werden Indikationen wie Schmerzen nach Verletzungen, schmerzhafte Schwellungen und Entzündungen nach Verletzungen oder mäßige bis starke Schmerzen ohne Spezifikation genannt, die einigen Interpretationsspielraum zulassen (Tab. 2). Die Expertengruppe ist sich einig, dass der perioperative Einsatz von NOPA mit ggf. auch schon prä- oder intraoperativer Gabe (siehe dazu auch Abschnitt 5) keinen Off-label-Use darstellt. NOPA sollten Bestandteil eines multimodalen perioperativen Analgesiekonzeptes (balancierte Analgesie) sein. Bei starken Schmerzen können NOPA mit Wirkstoffen anderer Substanzgruppen sowie weiteren medikamentösen und nichtmedikamentösen Verfahren kombiniert werden, wenn diese Kombination evidenzbasiert oder aufgrund einer Risiko-Nutzen-Abwägung sinnvoll ist. Übliche Einzel- und maximale Tagesdosen sowie Dosierungsintervalle für perioperativ häufig eingesetzte NOPA sind in Tab. 3 dargestellt.

**Table 2:** 

Substanz	Handelsnamen (Beispiele)	Applikation	Dosisintervall (h)	Max. Einzeldosis (mg)	Tagesdosis (mg)	Schmerzbezogene Indikation laut Fachinformation
Ibuprofen	Actren®, Dismenol®, Dolgit®, Esprenit®, Ibu-Lysin®, Imbun® Miralgin®, Nurofen-Saft®, Proff Schmerzkapseln®, Schmerz-Dolgit®, Thomapyrin®, Tispol Ibu DD®, TENSION® *Ibuprofen CT® Imbun®: 500 mg/ *1000 mg Filmtbl. **Actren Spezial® ***Dolormin®	p.o. supp.	8	400–800	1200–2400 bei KV-Risiko: 1200	Leichte bis mäßig starke Schmerzen, wie Kopfschmerzen, Zahnschmerzen, Regelschmerzen *Ausschließliche Indikationsformulierung bei rheumatischen Erkrankungen: vgl. Legende **Zusätzliche Indikation: akute Kopfschmerzen bei Migräne mit und ohne Aura ***Zusätzliche Indikation: Schmerzen bei Erkältung CH: u. a. postoperative Schmerzen und Schmerzen nach Dentaleingriffen
	Ibuprofen B. Braun	i.v.	6–8	600	1200	Kurzzeitbehandlung akuter mäßig starker Schmerzen
Diclofenac	*Diclo KD 75 mg akut®, Diclo CT®, Diclofenac AbZ 25 + 50 mg Tabletten®, Voltaren dispers® *Diclofenac 50 + 100 retard Heumann® ****Diclofenac Ratiopharm Lösung bei Migräne® ****Voltaren K Migräne 50 mg®	p.o. supp.	12	50–100 ret. 150	150	Schmerzhafte Schwellungen und Entzündungen nach Verletzungen und Operationen *Ausschließliche Indikationsformulierung bei rheumatischen Erkrankungen: vgl. Legende **Starke und sehr starke Schmerzen nach chirurgischen Eingriffen ***Weitere schmerzbezogene Indikationen: Tumorschmerz, Adnexitis, Dysmenorrhö ****Ausschließliche schmerzbezogene Indikation: Migräne
	*Diclofenac Ratiopharm 75 mg Injektionslösung®	i.v.	24	75	100–150^a^	Schmerzhafte Schwellungen und Entzündungen nach Verletzungen Schweiz postoperative + posttraumatische Schmerzen, Prophylaxe postoperativer Schmerzen *Weitere Indikationsformulierung: s. Legende
Dexketoprofen	Sympal 25 mg Lösung	p.o.	8	25	75	Kurzzeitige symptomatische Behandlung akuter Schmerzen von leichter bis mäßig starker Intensität, wie akute Schmerzen des Bewegungsapparates, Regelschmerzen, Zahnschmerzen
	Sympal Injekt 50 mg®	i.v.	8–12 (6)	50	150	Kurzzeitbehandlung mäßiger bis starker akuter Schmerzen, z. B. nach Operationen, bei Nierenkoliken, Rückenschmerzen, wenn eine Einnahme von Tabletten nicht geeignet ist
Naproxen	Proxen®	p.o.	12	500–750 max. 1000	1000	Schmerzen und Schwellungen nach chirurgischen Eingriffen Leichte bis mäßig starke Schmerzen, Schmerz und Entzündung bei rheumatischen Erkrankungen Leichte bis mäßig starke Schmerzen bei Kopf‑/Zahn‑/Regelschmerzen und bekannter Arthrose
	Naproxen Aristo®	p.o.	8–24	500–1000	500–1250	
	Aleve®	p.o.	8–12	400	600	
Celecoxib	Celebrex®	p.o.	12	100–200	400	Osteoarthritis, rheumatoide Arthritis, Spondylitis ankylosans
Etoricoxib	Arcoxia®	p.o.	24	60^b^ 90^c^ 120^d^	60–120 CH: 30–60	Postoperative Schmerzen nach Zahnoperationen Arthrose, rheumatoide Arthritis, Spondylitis ankylosans, akute Gichtarthritis ab 16 Jahre
Parecoxib	Dynastat®	i.v.	6–12	20–40	80	Kurzzeitbehandlung postoperativer Schmerzen bei Erwachsenen ab 18 Jahre
Metamizol	Novalgin® Novaminsulfon®	i.v.	4–6	1000^e^	5000	Akute starke Schmerzen nach Verletzungen und Operationen Sonstige akute oder chronische starke Schmerzen, soweit andere therapeutische Maßnahmen nicht indiziert sind Koliken Tumorschmerzen
	Novalgin®, Nopain®, Novaminsulfon®, Metamizol AbZ®, Metamizol Heumann®, Berlosin®, Baralgin®, Analgin®	p.o. supp.	4–6	1000	4000	
Paracetamol	Perfalgan® Paracetmol B Braun 10 mg/ml Infusionslösung®	i.v.	6	1000	4000	Kurzzeitbehandlung mäßig starker Schmerzen, besonders nach Operationen, wenn die i.v. Behandlung aufgrund dringend erforderlicher Schmerzbehandlung klinisch gerechtfertigt ist und/oder andere Arten der Anwendung nicht möglich sind
	Ben-u-ron®, Paracetamol Heumann®, Paracetamol Hormosan®	p.o. supp.	6	1000	4000	Symptomatische Behandlung leichter bis starker Schmerzen, Schmerzen nach Verletzungen

Empfehlungen zur Dosisreduktionen bei Komorbidität, eingeschränkter Organfunktion, hohem Alter bzw. bei Kindern und Jugendlichen werden nicht aufgeführt. Kombinationspräparate werden nicht aufgeführt

Ibuprofen, Diclofenac: *Ausschließliche Indikationsformulierung von Ibuprofen- und Diclofenac-Präparaten verschiedener Hersteller, überwiegend ohne direkte Angabe der Indikation Schmerz: akute und chronische Arthritiden, Spondylitis ankylosans und andere entzündlich-rheumatische Wirbelsäulenleiden, Arthrosen und Spondylarthrosen, Weichteilrheumatismus, schmerzhafte Schwellungen/Entzündungen nach Verletzungen

*KV* kardiovaskulär

^a^Diclofenac i.v. in Kombination mit weiteren Applikationsformen maximale Tagesdosis 150 mg

^b^Etoricoxib: Dosis bei der Indikation Arthrose

^c^Dosis für Indikation rheumatoide Arthritis und Schmerzen nach Zahnoperationen

^d^Dosis für Indikation akute Gichtarthritis

^e^Metamizol: bei strenger Indikationsstellung und besonders sorgfältiger Risiko-Nutzen-Abwägung kann die Einzeldosis auf 2500 mg erhöht werden

**Table 3:** 

Substanz	Initialdosis (mg)	Dosierungsintervall (h)	Tagesdosis (mg)	Maximale Anwendungsdauer laut Fachinformation
*Präoperative Gabe oral*				
Celecoxib	200	12	400	Kürzest möglich
Diclofenac	50–100	12	150	–
Etoricoxib	60–90 1 mg/kg	24	60–120	Möglichst kurzer Zeitraum Zahnoperation: 90 mg/Tag für maximal 3 Tage
Ibuprofen	600–800	6–8	2400	–
*Intraoperative Gabe i.v.*				
Dexketoprofen i.v.	50	(6) 8–12	150	Kurzzeitige Anwendung, nicht länger als 2 Tage
Ibuprofen i.v.	400–600	6–8	1200	Kürzest möglich, nicht länger als 3 Tage
Parecoxib i.v.	40	6–12	80	Kürzest möglich; >3 Tage nur begrenzte klinische Erfahrung
Metamizol i.v.	1000	4–6	5000	Dauer der Anwendung richtet sich nach Art und Schwere der Erkrankung
Paracetamol i.v.	1000	6	4000	Kurzzeitbehandlung

Dosisreduktion bei Komorbidität, älteren Patienten und Körpergewicht <50 kg entsprechend den Fachinformationen beachten

#### Empfehlung I. 1

NOPA sollten Bestandteil eines multimodalen perioperativen Analgesiekonzeptes sein.

Bei Operationen, die mit hoher Wahrscheinlichkeit mit stärkeren postoperativen Schmerzen verbunden sind und bei denen eine systemische Analgesie postoperativ geplant ist, können NOPA *prä- bzw. intraoperativ* unter Beachtung der Kontraindikationen gegeben werden.

### 2. NOPA und kardiovaskulären Vorerkrankungen

Welches NOPA primär eingesetzt wird, sollte anhand der Effektivität eines NOPA und des individuellen Risikos entschieden werden (Alter des Patienten, Komorbidität, Kontraindikationen, Art des operativen Eingriffes, Verfügbarkeit der Medikamente, auch in einer bestimmten Galenik, ob eine antiphlogistische Wirkung vorteilhaft ist etc.). Ibuprofen, Diclofenac und Naproxen haben in Abhängigkeit von Wirkstärke und Applikationsform unterschiedliche schmerzbezogene Indikationen. Viele Handelspräparate sind ausschließlich bei rheumatischen Erkrankungen zugelassen (Tab. 2). Dies gilt auch für Celecoxib, während Etoricoxib für maximal 3 Tage nach Zahnoperationen eine Zulassung in Deutschland hat. Die Präparate, für die explizit als Indikation die postoperative Analgesie bzw. Schmerzen nach Operationen und Verletzungen genannt werden, sind i.v. Paracetamol, i.v. Parecoxib, Metamizol sowie Dexketoprofen. Unabhängig davon haben NOPA jedoch seit Jahrzehnten Eingang in die perioperative Schmerztherapie gefunden und werden täglich zur perioperativen Analgesie bei der Mehrheit der Patienten eingesetzt [70].

In einer Cochrane-Analyse zur ***oralen Einmalgabe von Analgetika*** wurde die NNT („number needed to treat“) für diverse Analgetika berechnet, die zur Schmerzreduktion nach kleineren Operationen (mehrheitlich Zahnextraktionen sowie kleinere orthopädische und urologische Eingriffe, Episiotomien) eingesetzt wurden. Diese Zahl gibt an, wie viele Patienten behandelt werden müssen, damit bei einem Patienten eine über 4–6 h anhaltende mindestens 50 %ige Schmerzreduktion durch das Analgetikum gegenüber einer Placebobehandlung erreicht wird. Die NNT betrug für 50 mg Diclofenac 2,1 (1,9–2,5), für 500 mg Metamizol 2,4 (1,8–3,1), für 400 mg Ibuprofen 2,5 (2,4–2,6), für 400 mg Celecoxib 2,6 (2,3–3,0) und für 500 mg bzw. 1000 mg Paracetamol 3,5 (2,7–4,8) bzw. 3,6 (3,2–4,1; [36, 54, 55]). Für 90 mg bzw. 120 mg Etoricoxib wurde eine NNT von 1,7 (1,4–2,1) bzw. 1,8 (1,7–2,0) errechnet [19, 25].

Zu der üblichen Dosierung von 1 g Metamizol gibt es mangels Daten keine Angabe. Ebenso ist hervorzuheben, dass diese NNT in der Regel auf dem analgetischen Effekt einer oralen Einzeldosis nach einem kleinen chirurgischen Eingriff, meist Zahnextraktion in Lokalanästhesie, beruht. Dies ist nicht unbedingt auf die perioperative Anwendung nach größeren Operationen mit einem multimodalen Analgesieregime übertragbar.

Nach Ansicht der Expertengruppe kann Metamizol wie die anderen NOPA in der Akutschmerztherapie als Medikament der ersten Wahl eingestuft werden und ist hinsichtlich analgetischer Potenz wahrscheinlich vergleichbar mit NSAIDs, auch wenn die Studienlage nicht so umfangreich ist [45, 60, 64, 82, 91, 92].

Die intravenöse bzw. intramuskuläre Gabe einer Einzeldosis von Parecoxib im Vergleich zu Placebo nach gynäkologischen Laparoskopien (drei Studien), Kniegelenksersatz (eine Studie) und operativer Entfernung von mindestens zwei impaktierten Weisheitszähnen (drei Studien) wurde in einer Cochrane-Analyse untersucht. Es ergab sich eine NNT von 2,4 (2,1–2,8) für 20 mg und 1,8 (1,5–2,3) für 40 mg Parecoxib, um eine Schmerzreduktion von 50 % über 6 h zu erzielen [47]. In einem anderen Cochrane-Review wurde für Paracetamol bzw. Propacetamol (Prodrug von Paracetamol) vs. Placebo eine NNT von 6,0 (4,6–7,1) bzw. 5,0 (3,7–5,6) für eine mindestens 50 %ige Schmerzreduktion über 6 bzw. 4 h errechnet [53]. Im Vergleich i.v. Paracetamol vs. NSAID benötigten 34 % vs. 28 % zusätzliche Ausweichmedikation nach orthopädischen und gynäkologischen/geburtshilflichen Eingriffen (n. s., geringe Patientenzahlen) [53].

Auch wenn durch Paracetamol in einer Metaanalyse eine Reduktion des postoperativen Opioidbedarfs erzielt werden konnte, war diese meist weniger ausgeprägt als unter NSAIDs [20, 51]. Opioidbedingte Nebenwirkungen waren unter Paracetamol im Gegensatz zu NSAIDs nicht vermindert [20]. Im Vergleich zu NSAIDs erwies sich Paracetamol in diversen Studien als weniger analgetisch potent [8, 15, 27, 51, 54, 56, 67].

Die Expertengruppe stuft die analgetische Potenz traditioneller NSAIDs, der Coxibe und des Metamizols im perioperativen Setting als vergleichbar ein. Paracetamol wird als weniger potent eingeschätzt.

#### Kardiovaskuläre Vorerkrankungen und perioperative NSAIDs

Bei gesunden Patienten ohne kardiovaskuläre Risikofaktoren können sowohl traditionelle NSAIDs als auch Coxibe bei gegebener Indikation perioperativ zeitlich begrenzt eingesetzt werden. Bei Patienten mit kardiovaskulären Risikofaktoren (arterielle Hypertonie, Diabetes mellitus, Hyperlipidämie, Rauchen) und ohne weitere Kontraindikationen für NSAIDs kann nach Nutzen-Risiko-Abwägung im Einzelfall zeitlich begrenzt z. B. niedrig dosiertes Ibuprofen oder Celecoxib eingesetzt werden, nicht aber Diclofenac [78].

Bestehen hingegen *kardiovaskuläre ****Vorerkrankungen*** sind die in Tab. 1 angegebenen Gegenanzeigen und Warnhinweise zu beachten. Demnach sind bei Patienten mit Herzinsuffizienz NYHA II–IV alle NSAIDs kontraindiziert. Diclofenac und Coxibe dürfen darüber hinaus auch bei KHK, peripherer arterieller Verschlusskrankheit und zerebrovaskulären Erkrankungen nicht gegeben werden.

##### Empfehlung I. 2

Entsprechend der Gegenanzeige laut Fachinformation sollen Patienten mit kardiovaskulären Vorerkrankungen perioperativ keine NSAIDs erhalten.

Ein kurzfristiger Einsatz von NSAIDs kann jedoch bei Patienten mit kardiovaskulären Vorerkrankungen im Einzelfall eine Option sein, wenn Behandlungsalternativen fehlen und angenommen werden muss, dass unbehandelte Schmerzen hinsichtlich kardiovaskulärer Ereignisse evtl. noch problematischer sind. Aus medikolegaler Sicht kommt in solchen Einzelfällen, in denen trotz kardiovaskulärer Vorerkrankungen ein NSAID eingesetzt werden soll, neben der individuellen Nutzen-Risiko-Abwägung auch der** Patienteninformation mit einer Risikoaufklärung und der Darlegung möglicher Alternativen sowie einer sorgfältigen Dokumentation** eine hohe Bedeutung zu.

### 3. Kombination von NOPA

Zur Kombination von Paracetamol und einem NSAID ist die Studienlage heterogen. Zwei Metaanalysen zeigen für viele Studien eine Überlegenheit der Kombination von Paracetamol plus einem NSAID gegenüber einer alleinigen Gabe der Substanzen, andere Autoren sehen durch die Gabe von Paracetamol keinen zusätzlichen analgetischen Effekt [15, 24, 25, 34, 49, 57, 63, 95].

Mit einer Niedrigdosiskombination (200 mg Ibuprofen + 500 mg Paracetamol) nach Zahneingriffen konnte eine Analgesie und Opioideinsparung erzielt werden [24, 55]. Nach Kniegelenksersatz war jedoch Ibuprofen 400 mg + Paracetamol 1000 mg der Niedrigdosiskombination oder der alleinigen Gabe von Paracetamol überlegen. Ibuprofen 400 mg erwies sich als wirksamer als Paracetamol sowie die Niedrigdosiskombination und nicht deutlich schlechter als die Hochdosiskombination. Allerdings fehlt ein Vergleich zwischen der Kombination und der üblicherweise im perioperativen Setting eingesetzten Dosierungen von 600 bzw. 800 mg Ibuprofen. Ein niedrig bis moderat dosiertes NOPA, z. B. 400 mg Ibuprofen, das rezeptfrei in einer Apotheke erhältlich ist, kann oftmals nicht ausreichend sein, um perioperative suffiziente analgetische Effekte zu erzielen.

Die längerfristige Behandlung mit dieser NOPA-Kombination wird von einigen Autoren kritisch gesehen, da die durch Paracetamol ebenfalls induzierte COX-2-Inhibition bei einer Kombination mit einem NSAID mehr Nebenwirkungen bewirken kann [13, 26, 39].

Laut einer Umfrage wird die Kombination von Metamizol mit einem anderen NOPA in Deutschland häufig praktiziert: 47 % der antwortenden Anästhesisten kombinieren Metamizol mit einem traditionellen NSAID, 35 % mit Paracetamol und 33 % mit einem Coxib [70]. Aufgrund theoretischer Überlegungen scheint eine additive Wirkung von Metamizol plus einem anderen NOPA wahrscheinlich, allerdings liegen hierzu nur wenige randomisierte, kontrollierte Studien vor [49, 91, 97].

Hingegen sollten Patienten unter einem traditionellen NSAID bzw. Coxib in ausreichender Tagesdosis nicht zusätzlich noch ein anderes NSAID erhalten. Alle NSAIDs wirken über die Hemmung der COX‑2. Eine vollständige COX-Hemmung kann nicht weiter verstärkt werden. Daher ist die Kombination zweier Präparate dieser Substanzgruppe, jedes für sich in ausreichender Dosierung, nicht sinnvoll. Dieses würde nur das Risiko von Nebenwirkungen erhöhen [37]. Bei unzureichendem Ansprechen auf ein NSAID kann der Wechsel auf ein anderes NSAID hilfreich sein. Wegen interindividueller Unterschiede in Bioverfügbarkeit und Halbwertszeit kann die Wirksamkeit eines NSAID zwischen einzelnen Patienten erheblich differieren [11].

Ein eventueller Vorteil einer prä- und intraoperativen Gabe mehrerer NOPA unterschiedlicher Substanzgruppen wurde bis jetzt kaum systematisch untersucht, obwohl dieses Vorgehen in der klinischen Praxis weit verbreitet ist [70]. Aktuelle Registerdaten von über 1000 Patienten nach Rückenoperationen bestätigten eine bessere Analgesie bei intraoperativer Gabe von zwei oder sogar drei NOPA verschiedener Substanzgruppen (NSAID, Metamizol, Paracetamol) im Vergleich zu keinem oder nur einem NOPA [7]. Auch bei Kindern nach Tonsillektomien und Appendektomien war die intraoperative Gabe von mindestens zwei NOPA mit weniger Wunsch nach Schmerzmitteln für die ersten 24 h postoperativ assoziiert im Vergleich zu keinem oder einem NOPA [84]. Weitere qualitativ hochwertige Studien zu dieser Fragestellung sind notwendig.

#### Empfehlung I. 3

Im Rahmen eines perioperativen Analgesieregimes können NOPA verschiedener Substanzgruppen miteinander kombiniert werden.

## II. Präoperative Evaluation und Planung der Schmerztherapie

### 1. Anamnese

Die US-amerikanische Leitlinie zur Behandlung postoperativer Schmerzen adressiert in ihrer ersten Empfehlung die präoperative Patientenedukation und die Planung der perioperativen Schmerztherapie (US Guidelines: „strong recommendation“, „low-quality evidence“ [18]):

The panel recommends that clinicians provide patient and family-centered, individually tailored education to the patient (and/or responsible caregiver), including information on treatment options for management of postoperative pain, and document the plan and goals for postoperative pain management.

Im Rahmen der anästhesiologischen und chirurgischen Anamneseerhebung sollte auch zu vorbestehenden Schmerzen, Einnahme und Verträglichkeit von Analgetika gefragt werden. Kontraindikationen und mögliche Risikofaktoren, frühere Erfahrungen mit NOPA und deren Effektivität sind weitere hilfreiche Informationen. Handelsnamen der Analgetika (Tab. 2) sollten benannt werden, da Patienten die Substanznamen meist nicht kennen.

#### Empfehlung II. 1

Im Rahmen der anästhesiologischen und chirurgischen Anamnese und Aufklärung soll auch eine Schmerz- und Analgetikaanamnese durchgeführt werden.

### 2. Risikopatienten identifizieren

Im Rahmen der Prämedikation sollen Patienten mit einem erhöhten Risiko für starke postoperative Schmerzen sowie chronische Schmerzen nach Operation identifiziert werden. Häufig können solche Patienten mit einem Standardschema nicht suffizient behandelt werden. Risikofaktoren für starke postoperative Schmerzen sind u. a. präoperativ vorbestehende Schmerzen, eine vorbestehende Analgetikaeinnahme (vor allem Opioide), psychosoziale Faktoren wie Angst und Depressivität, jüngeres (Erwachsenen‑)Alter, weibliches Geschlecht und sozioökonomische Faktoren [3, 33, 38, 44, 100]. Auch hohe Schmerzerwartung, hohe Schmerzempfindlichkeit und das Katastrophisieren von Schmerzen wurden in einigen Studien als Prädiktoren beschrieben [69, 71, 98, 100]. Ähnliche Risikofaktoren konnten auch für die Chronifizierung postoperativer Schmerzen identifiziert werden (Entwicklung chronischer postoperativer Schmerzen; „chronic postsurgical pain“ [CPSP], siehe neue ICD-11-Definition). CPSP ist nach bestimmten Eingriffen besonders häufig [35]. Hierzu zählen u. a. Thorakotomien, Leistenhernienoperationen und Amputationen, aber auch Eingriffe wie ein Kniegelenksersatz oder Wirbelsäulenoperationen. Ein erhöhtes Risiko für CPSP haben vor allem Patienten, die wegen bestehender Schmerzen und funktioneller Beeinträchtigung operiert werden, ggf. sogar mit Opioiden eingestellt sind (z. B. Patienten zum Kniegelenksersatz bei lange vorbestehender Kniegelenksarthrose) [31, 85]. Für solche Konstellationen sollten zusammen mit der operativen Abteilung Klinikstandards entwickelt werden. Bei komplexen Fällen sollte frühzeitig ein Schmerzmediziner einbezogen werden.

#### Empfehlung II. 2

Im Rahmen der anästhesiologischen Anamnese und Aufklärung sollen Patienten mit einem Risiko für starke postoperative Schmerzen und einem Risiko für die Entwicklung chronischer postoperativer Schmerzen identifiziert werden.

### 3. Präoperative Planung der perioperativen Analgesie

Abhängig vom geplanten operativen Eingriff und den individuellen Bedürfnissen ist es sinnvoll, gemeinsam mit dem Patienten einen Behandlungsplan zur perioperativen Analgesie abzustimmen (z. B. Regionalanalgesie bei entsprechend geeigneten Eingriffen, systemische Analgesie, nichtmedikamentöse Verfahren; [14, 18]). Ein gut informierter Patient, der in die Planung, Entscheidungen und Umsetzung des Analgesiekonzeptes eingebunden wird, hat später weniger Schmerzen [58, 80]. Empfehlenswert ist eine standardisierte Vorgehensweise in einer abteilungsübergreifenden SOP („standard operating procedure“) zu hinterlegen, ergänzt durch eingriffsspezifische Besonderheiten.

#### Empfehlung II. 3

Im Rahmen der präoperativen Patientenaufklärung sollte die perioperative Analgesie in Zusammenarbeit mit dem Patienten geplant werden.

### 4. Präoperative Patienteninformation zu NOPA

In den letzten Jahren sind die potenziellen Risiken der NOPA in den Vordergrund der Diskussion gerückt. Die rechtliche Lage hinsichtlich der Aufklärungspflicht ist eindeutig (Bürgerliches Gesetzbuch [BGB] § 630c zu Informations- und Aufklärungspflichten des Arztes): „Der Behandelnde ist verpflichtet, dem Patienten in verständlicher Weise zu Beginn der Behandlung und, soweit erforderlich, in deren Verlauf sämtliche für die Behandlung wesentlichen Umstände zu erläutern, insbesondere … die Therapie und die zu und nach der Therapie zu ergreifenden Maßnahmen“. Daraus ergibt sich, dass **Patienten präoperativ über die perioperative Gabe von Analgetika und mögliche Nebenwirkungen aufzuklären sind.**

Dabei geht es um eine angemessene und realistische Nutzen-Risiko-Vermittlung. Noceboeffekte durch ein Übermaß an negativer und angsterzeugender Information sollten vermieden werden. Analgetika (NOPA und Opioide) sollten im Kontext der Medikamente für die Anästhesie und Analgesie benannt und in die Aufklärung über mögliche Komplikationen einbezogen werden.

Ein pragmatischer Ansatz wäre, die in der schriftlichen Einverständniserklärung zur Anästhesie schon vorhandenen Informationen zu möglichen Komplikationen entsprechend zu ergänzen. Die in jeder Anästhesieaufklärung genannten potenziell lebensbedrohlichen allergischen Reaktionen und kardiovaskulären Komplikationen können auch unter NOPA auftreten. Zu ergänzen wären Blutbildveränderungen (Agranulozytose), Schädigung des Magen-Darm-Trakts und der Nieren sowie der Leber. Da die perioperative Schmerztherapie fachgebietsübergreifend ist, sollten die Patienteninformation und -aufklärung sowie deren Dokumentation innerhalb eines Krankenhauses (Anästhesie, operative Abteilungen) vereinheitlicht werden.

#### Empfehlung II. 4

Die Patienteninformation und -aufklärung sollte das Nutzen-Risiko-Verhältnis der Gabe von NOPA und möglichen Alternativen, den Informationsbedarf des Patienten und einen möglichen „Noceboeffekt“ berücksichtigen.

## III. NOPA prä- und intraoperativ

### 1. Zeitpunkt der prä- und intraoperativen Gabe von NOPA

Die prä- und intraoperativ Gabe von NOPA ist in den Fachinformationen dieser Medikamente nicht explizit benannt. Vielmehr wird bei einigen NOPA allgemein von der Indikation „Kurzzeittherapie postoperativer Schmerzen“ gesprochen (Tab. 2).

Gemäß den Ergebnissen der schon zitierten Umfrage zur perioperativen Anwendung von NOPA geben 22 % der antwortenden Anästhesisten schon präoperativ, 86 % intraoperativ ein NOPA [70]. Somit ist die intraoperative Gabe von NOPA ein allgemein übliches Vorgehen in der Anästhesiologie. Auch in wissenschaftlichen Studien wurden NOPA vielfach schon vor Narkoseende appliziert bzw. diese frühe Gabe explizit empfohlen [12, 43, 45, 60, 91].

Eine Metaanalyse ergab, dass die präoperative Gabe von Celecoxib im Vergleich zu Placebo zu einer moderaten Abnahme des postoperativen Opioidbedarfs (−4,1 [95 %-CI: −5,6 bis −2,7] mg Morphin), der Schmerzintensität (NRS 0–10: −1,02 [−1,5 bis −0,5]) sowie von Übelkeit (−44 %) und Erbrechen (−38 %) führte, ohne vermehrte perioperative Blutungen und bei gleicher Patientenzufriedenheit [46].

Ob ein NOPA prä- oder intraoperativ gegeben wird, sollte unter Berücksichtigung der individuellen Kontraindikationen von der Operationsdauer und der Wirkdauer des Präparates abhängig gemacht werden. Bei einer geplanten Operationszeit von 4 h ist es für die postoperative Analgesie wenig hilfreich, Paracetamol oder Metamizol nach Narkoseeinleitung zu geben, da beide Analgetika nicht lang genug wirken. Sinnvoller wäre es in einem solchen Fall, diese Analgetika kurz vor Narkoseende zu infundieren. Ziel ist dabei nicht eine intraoperative Analgesie, sondern das Erreichen suffizienter Blutspiegel nach Narkoseausleitung. Dadurch können in der frühen postoperativen Phase starke Schmerzen, der Opioidverbrauch, ggf. auch opioidbedingte Nebenwirkungen reduziert werden [46]. Ist eine antiinflammatorische Wirkung erwünscht, kann präoperativ ein länger wirksames NSAID gegeben werden.

Hingegen ist bei typischen pädiatrischen Eingriffen mit relativ kurzer Operationszeit eine Analgetikagabe (NSAID oder Paracetamol als Suppositorium) nach Narkoseeinleitung und vor Schnitt tägliche Praxis.

#### Empfehlung III. 1

Die prä- bzw. intraoperative Gabe eines NOPA soll zeitlich so geplant werden, dass nach Ende einer Allgemeinanästhesie beim wachen Patienten analgetisch wirksame Plasmakonzentrationen erreicht werden.

Für die intraoperative Gabe kommen z. B. die intravenös applizierbaren Präparate Parecoxib, Metamizol, Paracetamol, Dexketoprofen und Ibuprofen infrage (Tab. 3). Ein orales NOPA kann bei nicht zu langer Operationsdauer auch vor Anästhesiebeginn gegeben werden, wie es z. B. in den US-Leitlinien empfohlen wird [18]. In einer Untersuchung war i.v. Parecoxib präoperativ oder bei Wundverschluss gegeben gleich wirksam. Beide Gruppen waren Placebo hinsichtlich Analgesie und zusätzlichem Opioidbedarf deutlich überlegen (66 % bzw. 72 % weniger PCA-Morphin nach Hüftgelenksersatz; [48]). Auch ein traditionelles NSAID kommt für die intra- oder präoperative Gabe unter Beachtung der Kontraindikationen infrage. Seine analgetische Wirkung wird vergleichbar der eines Coxibs eingeschätzt. Traditionelle NSAIDs verursachen jedoch eher gastrointestinale Nebenwirkungen, wenn keine entsprechende Prophylaxe bei gefährdeten Patienten gegeben wird, und erhöhen das Risiko für Blutungen.

### 2. Intraoperative Hypovolämie und Nierenfunktion

NSAIDs können bei Operationen mit größeren Blutverlusten und längeren hypotonen Kreislaufphasen zu Nierenfunktionsstörungen bis hin zum Nierenversagen führen. Die Gabe eines NSAIDs soll grundsätzlich erst erfolgen, wenn die Kreislaufverhältnisse stabilisiert worden sind und ein Volumenmangel ausgeglichen worden ist. Diese Kriterien können ggf. schon am Operationsende erfüllt sein oder auch erst im Verlauf der postoperativen Überwachung.

#### Empfehlung III. 2

Bei Operationen mit einem Risiko für größere Blutverluste soll – unabhängig von der Nierenfunktion – prä- bzw. intraoperativ kein NSAID gegeben werden.

Hingegen soll bei schon bekannter eingeschränkter Nierenfunktion (s. Fachinformationen) oder wenn durch den Eingriff eine Einschränkung der Nierenfunktion zu erwarten ist, perioperativ kein NSAID appliziert werden.

#### Empfehlung III. 3

Bei Patienten mit vorbestehender eingeschränkter Nierenfunktion soll generell kein NSAID perioperativ gegeben werden.

## IV. NOPA postoperativ

### 1. Anwendungsdauer der NOPA postoperativ

Die Therapie mit NOPA sollte nach der Operation fortgeführt werden, wenn eine postoperative Analgetikagabe notwendig ist. Zunächst sollten NOPA im Rahmen der Basisanalgesie in festen Zeitintervallen und ausreichender Dosierung gegeben werden (Tab. 3). Eine Umstellung auf eine orale Gabe sollte so bald wie möglich erfolgen.

Die Einnahme von NOPA (wie auch von Opioiden) soll von vornherein zeitlich befristet werden, um eine unkontrollierte Fortführung ohne fortbestehende Indikation zu vermeiden. Eine nur kurzfristige Anwendung mit niedriger Dosis senkt das Risiko für Nebenwirkungen. In den Fachinformationen wird für intravenöses Paracetamol eine Kurzzeittherapie angegeben, während für Parecoxib nur begrenzte klinische Erfahrung für eine längere Behandlungsdauer als drei Tage vorliegt. Auch die Gabe von Etoricoxib nach Zahnoperationen sollte laut Fachinformation auf drei Tage begrenzt sein. In der Metamizol-Fachinformation wird angegeben, dass sich die Dauer der Anwendung nach Art und Schwere der Erkrankung richtet: „So kurz wie möglich, so lange wie nötig“.

Nach größeren Operationen und anhaltenden Schmerzen kann es jedoch sinnvoll sein, die Einnahme von NOPA auch länger als drei Tage fortzuführen. Im Weiteren sollten zunächst die Opioide reduziert und dann abgesetzt werden und nachfolgend die NOPA.

#### Empfehlung IV. 1

NOPA sollen nur für einen begrenzten Zeitraum gegeben werden.

Hingegen erfordert das Fortführen einer analgetischen Medikation über einen für die entsprechende Operation üblichen Zeitraum hinaus eine klare Indikationsstellung und ggf. das Einbeziehen eines Schmerzmediziners.

### 2. Abteilungsübergreifender Behandlungsstandard mit NOPA

Für die sichere Anwendung perioperativer Analgetika bedarf es eines klinikübergreifenden Analgesiekonzeptes, das mit den beteiligten Disziplinen und Berufsgruppen abgestimmt und schriftlich, z. B. als abteilungsübergreifender Behandlungsstandard/SOP/Klinikalgorithmus, niedergelegt wird. Darin soll eine Beschränkung auf einige wenige NOPA in adäquater Dosierung und für adäquate Applikationszeiträume erfolgen und klar formuliert werden, welches NOPA das Medikament der ersten, zweiten bzw. dritten Wahl ist und welche Kombinationen gegeben werden können. Diese Festlegung sollte ggf. auch abgestimmt sein auf die Anforderungen spezifischer Eingriffe und Patientenprofile. Auch zu den Verantwortlichkeiten der beteiligten Fachdisziplinen sollte in einem Krankenhaus Konsens hergestellt und dieser schriftlich fixiert werden [14]. Nur durch solche transparenten, für alle beteiligten Disziplinen und Professionen einsehbaren Behandlungsstandards lassen sich Unsicherheiten bei der Anwendung vermeiden, Dosierungsfehler und Verwechslungen minimieren und der sinnvolle Einsatz von Analgetika‑/Medikamentenkombinationen und weiterer (analgetischen) Maßnahmen ermöglichen.

#### Empfehlung IV. 2

Die Therapie mit NOPA soll in einem abteilungsübergreifenden Behandlungsstandard hinterlegt werden. Es soll ein Konzept mit NOPA der ersten, zweiten und dritten Wahl und die Umstellung von einer festen Tagesdosis auf eine Bedarfsmedikation bis zum Absetzen der Analgetika festgelegt werden.

### 3. Information an den nachbehandelnden Arzt

Bei zunehmend kürzerer Krankenhausverweildauer sowie vermehrt ambulant durchgeführten Eingriffen verschiebt sich die postoperative Schmerztherapie zunehmend auf den Zeitraum nach der Krankenhausentlassung.

Vor der Entlassung soll die weitere Verschreibung von Analgetika kritisch geprüft und nicht mehr indizierte Analgetika sollen abgesetzt werden. Bei noch bestehendem Analgetikabedarf ist eine zeitlich befristete Verschreibung mit Information an den nachbehandelnden Arzt/Hausarzt sinnvoll. Der nachbehandelnde Arzt soll im (operativen) Entlassungsbrief und dem Medikamentenplan über die perioperativ erfolgte Analgetikatherapie, die nach Entlassung fortzuführenden Medikamente/Analgetika und die geplante Dosisreduktion bis zum Absetzen der Analgetika informiert werden. Die Zuständigkeit zwischen den Abteilungen sollte geklärt werden, und der Entlassungsbrief der operativen Abteilung sollte ggf. auch den Hinweis auf eine vom Schmerzdienst spezifisch auf einen Patienten zugeschnittene Medikation umfassen. Die Weitergabe der Information auch zu gerade beendeter Medikation ist erforderlich, da z. B. eine Agranulozytose noch bis zu zehn Tage nach Absetzen des Metamizol auftreten kann [42, 86, 89].

#### Empfehlung IV. 3

Bei Entlassung aus dem Krankenhaus soll der nachbehandelnde Arzt zu perioperativ gegebenen und aktuell noch eingenommenen Analgetika schriftlich informiert werden.

Der nachbehandelnde Arzt sollte auf den temporären Charakter der perioperativen Schmerzmedikation aufmerksam gemacht werden. Länger anhaltende postoperative Schmerzzustände können Symptome postoperativer Komplikationen und/oder der Entwicklung chronischer postoperativer Schmerzen sein und sollten zügig abgeklärt werden.

### 4. Informationspflicht des Arztes

Patienten mit beendeter oder noch laufender Analgetikatherapie sollen über mögliche Komplikationen und klinische Symptome (z. B. gastrointestinale Beschwerden, Möglichkeit der Abhängigkeit bei Opioiden), die bei weiterer Einnahme oder ggf. auch erst nach Absetzen des Medikaments (z. B. Agranulozytose) auftreten können, informiert werden [86, 87, 89]. Dafür ist eine adäquate Aufklärung zu Symptomen und ein entsprechendes Verhalten des Patienten erforderlich (Sicherungsaufklärung im Rahmen der Aufklärungs- und Informationspflicht des Arztes), um die Patienten für mögliche Komplikationen zu sensibilisieren und ihnen angemessene Verhaltensweisen aufzuzeigen. Solche therapeutischen Beratungen (Sicherungsaufklärungen) über mögliche Folgen einer Therapie und entsprechende Verhaltensweisen sind von anderen Verfahren bekannt [87]. Auch dieses kann nur in einem klinikübergreifenden Konzept zusammen mit den operativen Abteilungen etabliert werden, denn die Entlassung der Patienten mit entsprechendem Entlassungsbrief erfolgt in der Regel durch den (chirurgischen) Stationsarzt.

#### Empfehlung IV. 4

Patienten sollen zu möglichen Nebenwirkungen der Analgetika und ihrer Symptome, die auch nach Krankenhausentlassung auftreten können, und entsprechenden Verhaltensweisen informiert werden.

## Zusammenfassung der Empfehlungen

### I. Allgemeine Empfehlungen

1. NOPA sollten Bestandteil eines multimodalen perioperativen Analgesiekonzeptes sein.
  Bei Operationen, die mit hoher Wahrscheinlichkeit mit stärkeren postoperativen Schmerzen verbunden sind und bei denen eine systemische Analgesie postoperativ geplant ist, können NOPA ***prä- bzw. intraoperativ*** unter Beachtung der Kontraindikationen gegeben werden.
2. Entsprechend der Gegenanzeige laut Fachinformation sollen Patienten mit kardiovaskulären Vorerkrankungen perioperativ keine NSAID erhalten.
3. Im Rahmen eines perioperativen Analgesieregimes können NOPA verschiedener Substanzgruppen miteinander kombiniert werden.

### II. Präoperative Evaluation und Planung der Schmerztherapie

1. Im Rahmen der anästhesiologischen und chirurgischen Anamnese und Aufklärung soll auch eine Schmerz- und Analgetikaanamnese durchgeführt werden.
2. Im Rahmen der anästhesiologischen Anamnese und Aufklärung sollen Patienten mit einem Risiko für starke postoperative Schmerzen und einem Risiko für die Entwicklung chronischer postoperativer Schmerzen identifiziert werden.
3. Im Rahmen der präoperativen Patientenaufklärung sollte die perioperative Analgesie in Zusammenarbeit mit dem Patienten geplant werden.
4. Die Patienteninformation und -aufklärung sollte das Nutzen-Risiko-Verhältnis der Gabe von NOPA und möglicher Alternativen, den Informationsbedarf des Patienten und einen möglichen „Noceboeffekt“ berücksichtigen.

### III. NOPA prä- und intraoperativ

1. Die prä- bzw. intraoperative Gabe eines NOPA soll zeitlich so geplant werden, dass nach Ende einer Allgemeinanästhesie beim wachen Patienten analgetisch wirksame Plasmakonzentrationen erreicht werden.
2. Bei Operationen mit einem Risiko für größere Blutverluste soll – unabhängig von der Nierenfunktion – prä- bzw. intraoperativ kein NSAID gegeben werden.
3. Bei Patienten mit vorbestehender eingeschränkter Nierenfunktion soll generell kein NSAID perioperativ gegeben werden.

### IV. NOPA postoperativ

1. NOPA sollen nur für einen begrenzten Zeitraum gegeben werden.
2. Die Therapie mit NOPA soll in einem abteilungsübergreifenden Behandlungsstandard hinterlegt werden. Es soll ein Konzept mit NOPA der ersten, zweiten und dritten Wahl und die Umstellung von einer festen Tagesdosis auf eine Bedarfsmedikation bis zum Absetzen der Analgetika festgelegt werden.
3. Bei Entlassung aus dem Krankenhaus soll der nachbehandelnde Arzt zu perioperativ gegebenen und aktuell noch eingenommenen Analgetika schriftlich informiert werden.
4. Patienten sollen zu möglichen Nebenwirkungen der Analgetika und ihrer Symptome, die auch nach Krankenhausentlassung auftreten können, und entsprechenden Verhaltensweisen informiert werden.

### Supplementary Information



## Anhang zur Konsensstärke

Alle Empfehlungen erzielten >80 % Zustimmung (15/18 Stimmen). Empfehlungen, bei denen es keine 100 % Zustimmung gab, sind im Folgenden aufgeführt:

- I.1. erster Teil: ein Votum für „können“; ein Votum für „sollen“
- I.1. zweiter Teil drei Voten für „sollten“
- I.2. drei Voten für „sollten“
- I.3. ein Votum für „sollte“
- II.1. ein Votum für „sollte“
- II.2. zwei Voten für „sollten“
- III.1. drei Voten für „sollte“
- III.2. zweiter Teil: zwei Voten für „sollte“
- IV.2. erster Teil ein Votum für „sollte“
- IV.2. zweiter Teil: zwei Voten für „sollte“
- IV.3. ein Votum für „sollte“
- IV.4. zwei Voten für „sollten“
