# Supplementary material for: Perioperative analgesia with nonopioid analgesics: Joint interdisciplinary consensus-based recommendations of the German Pain Society, the German Society of Anaesthesiology and Intensive Care Medicine and the German Society of Surgery
Source: Chirurg. 2021 May 26;92(7):647–63. [Article in German] doi: 10.1007/s00104-021-01421-w (PMC8241738; doi:10.1007/s00104-021-01421-w)
Supplement: Supplementary file 1 [file 104_2021_1421_MOESM1_ESM.pdf]

Erklärung von Interessen

Perioperative Schmerztherapie mit Nicht-Opioid-Analgetika
Konsensbasierte Empfehlungen

zu Händen von Prof. Dr. Ulrike Stamer

Vorbemerkung

Alle Mitglieder der Leitliniengruppe sind gehalten, die nachstehende Erklärung von Interessen auszufüllen. Die Erklärung wird gegenüber dem Leitlinienkoordinator abgegeben. Dies soll bereits zu Beginn des Leitlinienprojekts erfolgen bzw. zu dem Zeitpunkt, an dem die Mitglieder ihre Teilnahme am Leitlinienprojekt gegenüber dem Koordinator bestätigen. Bei länger andauernden Projekten ist eine Erneuerung der Erklärung einmal pro Jahr bis zum Abschluss der Leitlinienentwicklung, zumindest aber vor der Konsensfindung, erforderlich.

In der Erklärung sind alle Interessen aufzuführen, unabhängig davon, ob der/die Erklärende selbst darin einen thematischen Bezug zur Leitlinie oder einen Interessenskonflikt sieht oder nicht. Ob Interessenkonflikte bestehen und ob dadurch die erforderliche Neutralität für die Mitarbeit bei der Leitlinienentwicklung in Frage gestellt ist oder in welchen speziellen Bereichen /Fragestellungen der Leitlinie das professionelle Urteilsvermögen eines Experten durch sekundäre Interessen beeinflusst sein könnte, ist durch einen Dritten zu bewerten und in der Leitliniengruppe zu diskutieren. Die Erklärung betrifft Interessen innerhalb **des laufenden Jahres sowie der zurückliegenden 3 Jahre.**

Die Originale der Erklärungen verbleiben vertraulich beim Leitlinienkoordinator. Die Inhalte der Erklärungen sind in der Langfassung der Leitlinie bzw. im Leitlinienreport in standardisierter Zusammenfassung offen darzulegen. Ergänzend sind das Verfahren der Sammlung und Bewertung der Erklärungen sowie die Ergebnisse der Diskussion zum Umgang mit Interessenkonflikten darzulegen.

<u>Informationen zur Datenerhebung gemäß Artikel 13 DSGVO</u>

Der Leitlinienkoordinator erhebt Ihre Daten zum Zweck des o.g. Leitlinienvorhabens sowie zur Erfüllung des Regelwerkes der AWMF. Die Datenerhebung und Datenverarbeitung sind für die Durchführung des Leitlinienvorhabens erforderlich und beruhen auf Artikel 6 Abs. 1 b) DSGVO. Eine Weitergabe der Daten an Dritte findet nur zum Zweck der Erfüllung des Regelwerkes der AWMF statt. Die Daten werden gelöscht, sobald sie für den Zweck ihrer Verarbeitung nicht mehr erforderlich sind. Sie sind berechtigt, Auskunft der im Rahmen des Leitlinienvorhabens über Sie gespeicherten Daten zu beantragen sowie bei Unrichtigkeit der Daten die Berichtigung oder bei unzulässiger Datenspeicherung die Löschung der Daten zu fordern.

Erklärung

1. Allgemeine Angaben

Name, Vorname, Titel	Stamer, Ulrike; Prof. Dr. med-	
Arbeitgeber / Institution	Klinik für Anästhesiologie und Schmerztherapie, Inselspital, Universität Bern	Früher(e) innerhalb des laufenden Jahres oder der 3 Kalenderjahre davor
Position / Funktion in der Institution	Oberärztin	
Adresse	Freiburgstrasse, 3010 Bern	
e-mail-Adresse	Ulrike.stamer@dbmr.unibe.ch	
Bei Rückfragen telefonisch zu erreichen unter	+41-31-6329995	
Funktion in der Leitliniengruppe	Koordinatorin	
Datum	20.2.2020	
Zeitraum, auf den sich die Erklärung bezieht	2017-2020	

2. Direkte, finanzielle Interessen

Hier werden finanzielle Beziehungen zu Unternehmen, Institutionen oder Interessenverbänden im Gesundheitswesen erfasst. Haben Sie oder die Einrichtung, für die Sie tätig sind, innerhalb des laufenden Jahres oder der 3 Kalenderjahre davor Zuwendungen erhalten von Unternehmen der Gesundheitswirtschaft (z.B. Arzneimittelindustrie, Medizinproduktindustrie), industriellen Interessenverbänden, kommerziell orientierten Auftragsinstituten, Versicherungen/Versicherungsträgern, oder von öffentlichen Geldgebern (z.B. Ministerien), Körperschaften/Einrichtungen der Selbstverwaltung, Stiftungen, oder anderen Geldgebern? Machen Sie bitte in folgender Tabelle zu allen zutreffenden Aspekten konkrete Angaben.

Art der Beziehung/Tätigkeit	Name des/der Kooperations-partner/s	Zeitraum der Beziehung/ Tätigkeit[1]	Thema, Bezug zur Leitlinie[2]	Art der Zuwendung[3]	Höhe der Zuwendung[4]	Empfänger [5]
Berater-/Gutachtertätigkeit	Gemeinsamer Bundesausschuss, Berlin	2017-2020		Reisekosten,. Aufwandsent-schädigung		persönlich
Mitarbeit in einem Wissenschaft-lichen Beirat (advisory board)						
Vortrags-/oder Schulungstätigkeit	Grünenthal, Schweiz	2/2018	Projektvorstellung PAIN OUT / keine	Aufwandsent-schädigung	500 CHF	Institution
Vortrags-/oder Schulungstätigkeit	Sanofi Aventis, Schweiz	9/2021	Vortrag Agranulo-zytose+ Metamizol /keine	Reisekosten, Aufwandsent-schädigung	560 CHF	Institution
Vortrags-/oder Schulungstätigkeit	Syntetica	10/2017	Vortrag Hydro-morphon / keine	Reisekosten, Auf-wandsent-schädi-gung	1000 CHF	Institution

[1] Innerhalb des Erfassungszeitraums, d.h.im gegenwärtigen und den zurückliegenden 3 Jahren, Angabe: von (Monat/Jahr) bis (Monat/Jahr)
[2] Angabe des Themas, bei Präparaten/Geräten auch Handelsname bzw. Wirkstoffname (Freitext), zusätzlich Angabe einer Selbsteinschätzung des Bezugs zur Leitlinie: „Nein“ oder „Ja“
[3] Honorar, Drittmittel, geldwerte Vorteile (z.B. Personal-oder Sachmittel; Reisekosten, Teilnahmegebühren, Bewirtung i.R. von Veranstaltungen), Verkaufslizenz
[4] Es können gerundete Beträge angeben werden (z.B. bei Beiträgen > 1000 € jeweils auf die nächste Tausenderstelle): Die Angaben beziehen sich auf die Gesamtsumme der Zuwendungen für eine angegebene Tätigkeit über den Erfassungszeitraum, Angabe: von (Monat/Jahr) bis (Monat/Jahr).
Diese Angaben werden vertraulich behandelt.
[5] Bitte angeben: a) wenn Sie persönlich Empfänger der Zuwendung sind oder b) wenn es die Institution ist, für die Sie tätig sind und Sie innerhalb Ihrer Institution direkt entscheidungsverantwortlich für die Verwendung der Zuwendung/Mittel sind. Sind Sie nicht direkt entscheidungsverantwortlich, sind keine Angaben nötig.

Vortrags-/oder Schulungstätigkeit	Deutsche Schmerz-gesellschaft, DGAI, SGAR	jährlich	Schmerztherapie	Reisekosten, ggf. Hotel		persönlich
Autoren-/oder Coautorenschaft	Siehe PubMed				keine	
Forschungsvorhaben/ Durchführung klinischer Studien<br><br>PROMPT IMI Pain Care EU und Pharma<br><br>PREVENT	PAIN OUT Universität Jena und EFIC<br><br>Uni Jena Schweizer Multizenter-studie, RCT<br><br>PI B. Rehberg-Klug, Genf	2018-2020<br><br>2019-2021<br><br>läuft noch	Verbesserung postoperative Schmerztherapie / kein Bezug<br><br>Verbesserung post-operative Analgesie	-<br><br>Aufwandsentschädi-gung für eingeschlos-sene Patienten	2 x Jahresgebühr PAIN OUT je 1500 € wurde von EFIC bezahlt<br><br>Keine<br><br>keine	Institution<br><br>Institution
Eigentümerinteressen (Patent, Urheberrecht, Aktienbesitz[6])	keine					

3. Indirekte Interessen

Hier werden persönliche Beziehungen zu Interessenverbänden im Gesundheitswesen, „intellektuelle“, akademische, und wissenschaftliche Interessen oder Standpunkte sowie Schwerpunkte klinischer Tätigkeiten/Einkommensquellen erfasst (für den Zeitraum des laufenden Jahres oder der 3 Kalenderjahre davor). Hierunter fallen auch solche, die indirekt mit finanziellen persönlichen Interessen verbunden sein können.

- Sind oder waren Sie in Wissenschaftlichen Fachgesellschaften, Berufsverbänden, Institutionen der Selbstverwaltung, Patientenselbsthilfegruppen, Verbrauchervertretungen oder anderen Verbänden aktiv? Wenn ja, in welcher Funktion (z.B. Mandatsträger für diese/andere Leitlinien, Vorstand)?
- Können Sie Schwerpunkte Ihrer wissenschaftlichen und /oder klinischen Tätigkeiten benennen? Fühlen Sie sich bestimmten „Schulen“ zugehörig?
- Waren Sie an der inhaltlichen Gestaltung von Fortbildungen federführend beteiligt?
- Haben Sie persönliche Beziehungen (als Partner oder Verwandter 1. Grades) zu einem Vertretungsberechtigten eines Unternehmens der Gesundheitswirtschaft?

Machen Sie bitte in folgender Tabelle zu allen zutreffenden Aspekten konkrete Angaben.

[6] Betrifft nur Eigentümerinteressen im Gesundheitswesen; auch sind Angaben zu Mischfonds nicht erforderlich.

Art der Beziehung/Tätigkeit	Namen / Schwerpunkte (bitte konkret benennen)	Zeitraum der Beziehung/ Tätigkeit[7]	Themenbezug zur Leitlinie[8]
Mitgliedschaft /Funktion in Interessenverbänden	DGAI, Mitglied<br>Wissenschaftlicher AK Schmerzmedizin der DGAI: Mitglied<br>Wissenschaftlicher AK Geburtshilfliche Anästhesie der DGAI: Mitglied<br>Deutsche Schmerzgesellschaft, 1. Sprecherin des AK Akutschmerz der Deutschen Schmerzgesellschaft<br>Schweizer Anästhesiegesellschaft (SGAR);<br>Schweizer Akutschmerz AG in der SGAR<br>International Association for the Study of Pain (IASP), USA<br>Special Interest Group 'Acute Pain' of the IASP: Mitglied<br>European Society of Anaesthesiology and Intensive Care (ESAIC): member<br>Sub-committee "Acute and Chronic Pain and Palliative Medicine" of the ESAIC: chair person<br>European Pain Federation (EFIC); member	alles länger als 10 Jahre und noch bestehend	
Schwerpunkte wissenschaftlicher Tätigkeiten, Publikationen	Postoperative Schmerztherapie, chronischer Schmerz nach Operationen, CPSP, genetische Prädisposition Schmerz + PONV		
Schwerpunkte klinischer Tätigkeiten	Schmerztherapie		
Federführende Beteiligung an Fortbildungen/Ausbildungsinstituten	ESAIC Chairperson Subcommittee Acute and chronic pain and palliative care<br>DGSS Sprecherin AK Akutschmerz<br>Seeheimer Akutschmerztage der Deutschen Schmerzgesellschaft.		
Persönliche Beziehungen (als Partner oder Verwandter 1. Grades) zu einem Vertretungsberechtigten eines Unternehmens der Gesundheitswirtschaft	nein		

[7] Innerhalb des Erfassungszeitraums, d.h. im gegenwärtigen und den zurückliegenden 3 Jahren, Angabe: von (Monat/Jahr) bis (Monat/Jahr)
[8] Angabe einer Selbsteinschätzung „Nein“ oder „Ja“

4. Sonstige Interessen

Sehen Sie andere Aspekte oder Umstände, die von Dritten als einschränkend in Bezug auf Ihre Objektivität oder Unabhängigkeit wahrgenommen werden könnten?
nein

Ich erkläre hiermit nach bestem Wissen und Gewissen, dass ich alle mir derzeit bekannten Umstände aufgeführt habe, die gegebenenfalls zu einem persönlichen Interessenkonflikt bei der themenbezogenen Mitwirkung bei der Erstellung der Leitlinie führen können. Ich erkläre weiterhin, dass ich die Diskussion der Erklärungen anderer Mitglieder in der Leitliniengruppe absolut vertraulich behandeln werde. Ich bin darüber informiert, dass die Angaben in standardisierter Zusammenfassung mit der Leitlinie/in einem begleitenden Leitlinienreport veröffentlicht werden, und dass das vorliegende Formular vor der Einsicht unberechtigter Dritter geschützt aufbewahrt wird. Hiermit bin ich einverstanden.

U. Stamer

20.2.2020

Datum **Unterschrift**

Ergänzende Hinweise

- Bitte füllen Sie das Formular vollständig aus.
- Falls Sie zu bestimmten Fragen keine Angaben machen können oder wollen, begründen Sie dies bitte.
- Bitte speichern Sie das ausgefüllte Formular und senden es an ulrike.stamer@dbmr.unibe.ch

AWMF

Erklärung von Interessen

Perioperative Schmerztherapie mit Nicht-Opioid-Analgetika Konsensbasierte Empfehlungen

zu Händen von Prof. Dr. Ulrike Stamer

Vorbemerkung

Alle Mitglieder der Leitliniengruppe sind gehalten, die nachstehende Erklärung von Interessen auszufüllen. Die Erklärung wird gegenüber dem Leitlinienkoordinator abgegeben. Dies soll bereits zu Beginn des Leitlinienprojekts erfolgen bzw. zu dem Zeitpunkt, an dem die Mitglieder ihre Teilnahme am Leitlinienprojekt gegenüber dem Koordinator bestätigen. Bei länger andauernden Projekten ist eine Erneuerung der Erklärung einmal pro Jahr bis zum Abschluss der Leitlinienentwicklung, zumindest aber vor der Konsensfindung, erforderlich.

In der Erklärung sind alle Interessen aufzuführen, unabhängig davon, ob der/die Erklärende selbst darin einen thematischen Bezug zur Leitlinie oder einen Interessenskonflikt sieht oder nicht. Ob Interessenkonflikte bestehen und ob dadurch die erforderliche Neutralität für die Mitarbeit bei der Leitlinienentwicklung in Frage gestellt ist oder in welchen speziellen Bereichen /Fragestellungen der Leitlinie das professionelle Urteilsvermögen eines Experten durch sekundäre Interessen beeinflusst sein könnte, ist durch einen Dritten zu bewerten und in der Leitliniengruppe zu diskutieren. Die Erklärung betrifft Interessen innerhalb **des laufenden Jahres sowie der zurückliegenden 3 Jahre.**

Die Originale der Erklärungen verbleiben vertraulich beim Leitlinienkoordinator. Die Inhalte der Erklärungen sind in der Langfassung der Leitlinie bzw. im Leitlinienreport in standardisierter Zusammenfassung offen darzulegen. Ergänzend sind das Verfahren der Sammlung und Bewertung der Erklärungen sowie die Ergebnisse der Diskussion zum Umgang mit Interessenkonflikten darzulegen.

Informationen zur Datenerhebung gemäß Artikel 13 DSGVO
Der Leitlinienkoordinator erhebt Ihre Daten zum Zweck des o.g. Leitlinienvorhabens sowie zur Erfüllung des Regelwerkes der AWMF. Die Datenerhebung und Datenverarbeitung sind für die Durchführung des Leitlinienvorhabens erforderlich und beruhen auf Artikel 6 Abs. 1 b) DSGVO. Eine Weitergabe der Daten an Dritte findet nur zum Zweck der Erfüllung des Regelwerkes der AWMF statt. Die Daten werden gelöscht, sobald sie für den Zweck ihrer Verarbeitung nicht mehr erforderlich sind. Sie sind berechtigt, Auskunft der im Rahmen des Leitlinienvorhabens über Sie gespeicherten Daten zu beantragen sowie bei Unrichtigkeit der Daten die Berichtigung oder bei unzulässiger Datenspeicherung die Löschung der Daten zu fordern.

Erklärung

1. Allgemeine Angaben

Name, Vorname, Titel	Erlenwein, Joachim, PD Dr. med.	
Arbeitgeber / Institution	Gegenwärtig<br>Universitätsmedizin Göttingen, Klinik für Anästhesiologie	Früher(e) innerhalb des laufenden Jahres oder der 3 Kalenderjahre davor<br>kein anderer Arbeitgeber in den letzten 3 Jahren
Position / Funktion in der Institution	Oberarzt der Klinik für Anästhesiologie (Schmerzmedizin - Schmerztagesklinik und Ambulanz und bereichsleitender Oberarzt für den innerklinischen Schmerzdienst)	
Adresse	Robert-Koch-Str. 40, 37075 Göttingen	
e-mail-Adresse	joachim.erlenwein@med.uni-goettingen.de	
Bei Rückfragen telefonisch zu erreichen unter	0551-39-12206	
Funktion in der Leitliniengruppe	Co-Autor, Vertreter WAK Schmerzmedizin, Deutsche Gesellschaft für Anästhesiologie und Intensivmedizin e.V. (DGAI, Nürnberg); Vertreter AK Akutschmerz, Deutsche Schmerzgesellschaft e.V.	
Datum	25.06.2020	
Zeitraum, auf den sich die Erklärung bezieht	jetzt, bezogen auf die vergangenen 3 Jahre	

2. Direkte, finanzielle Interessen

Hier werden finanzielle Beziehungen zu Unternehmen, Institutionen oder Interessenverbänden im Gesundheitswesen erfasst. Haben Sie oder die Einrichtung, für die Sie tätig sind, innerhalb des laufenden Jahres oder der 3 Kalenderjahre davor Zuwendungen erhalten von Unternehmen der Gesundheitswirtschaft (z.B. Arzneimittelindustrie, Medizinproduktindustrie), industriellen Interessenverbänden, kommerziell orientierten Auftragsinstituten, Versicherungen/Versicherungsträgern, oder von öffentlichen Geldgebern (z.B. Ministerien), Körperschaften/Einrichtungen der Selbstverwaltung, Stiftungen, oder anderen Geldgebern? Machen Sie bitte in folgender Tabelle zu allen zutreffenden Aspekten konkrete Angaben.

Art der Beziehung/Tätigkeit	Name des/der Kooperations-partner/s	Zeitraum der Beziehung/ Tätigkeit[1]	Thema, Bezug zur Leitlinie[2]	Art der Zuwendung[3]	Höhe der Zuwendung[4]	Empfänger [5]
Berater-/Gutachtertätigkeit	-	-	-	-	-	
Mitarbeit in einem Wissenschaft-lichen Beirat (advisory board)	-	-	-	-	-	
Vortrags-/oder Schulungstätigkeit	-	-	-	-	-	
Autoren-/oder Coautorenschaft	-	-	-	-	-	
Forschungsvorhaben/ Durchführung klinischer Studien	2019 bewilligter Antrag „POET PAIN“ der Deutschen	Mitarbeit an dem Projekt seit 2018	keine direkte	finanzielle Forsuchungs unterstützung	noch nicht exakt feststehen (Bescheid nach DLR Prüfung ausstehend)	Universitäts medizin Göttingen als Konsortialpa

[1] Innerhalb des Erfassungszeitraums, d.h.im gegenwärtigen und den zurückliegenden 3 Jahren, Angabe: von (Monat/Jahr) bis (Monat/Jahr)
[2] Angabe des Themas, bei Präparaten/Geräten auch Handelsname bzw. Wirkstoffname (Freitext), zusätzlich Angabe einer Selbsteinschätzung des Bezugs zur Leitlinie: „Nein“ oder „Ja“
[3] Honorar, Drittmittel, geldwerte Vorteile (z.B. Personal-oder Sachmittel; Reisekosten, Teilnahmegebühren, Bewirtung i.R. von Veranstaltungen), Verkaufslizenz
[4] Es können gerundete Beträge angeben werden (z.B. bei Beiträgen > 1000 € jeweils auf die nächste Tausenderstelle): Die Angaben beziehen sich auf die Gesamtsumme der Zuwendungen für eine angegebene Tätigkeit über den Erfassungszeitraum, Angabe: von (Monat/Jahr) bis (Monat/Jahr).
Diese Angaben werden vertraulich behandelt.
[5] Bitte angeben: a) wenn Sie persönlich Empfänger der Zuwendung sind oder b) wenn es die Institution ist, für die Sie tätig sind und Sie innerhalb Ihrer Institution direkt entscheidungsverantwortlich für die Verwendung der Zuwendung/Mittel sind. Sind Sie nicht direkt entscheidungsverantwortlich, sind keine Angaben nötig.

	Schmerzgesellschaft beim Innovationsfonds des Gemeinsamen Bundesausschuss					rtner, vertreten durch J. Erlenwein
Eigentümerinteressen (Patent, Urheberrecht, Aktienbesitz[6])	-	-	-	-		

[6] Betrifft nur Eigentümerinteressen im Gesundheitswesen; auch sind Angaben zu Mischfonds nicht erforderlich.

3. Indirekte Interessen

Hier werden persönliche Beziehungen zu Interessenverbänden im Gesundheitswesen, „intellektuelle", akademische, und wissenschaftliche Interessen oder Standpunkte sowie Schwerpunkte klinischer Tätigkeiten/Einkommensquellen erfasst (für den Zeitraum des laufenden Jahres oder der 3 Kalenderjahre davor). Hierunter fallen auch solche, die indirekt mit finanziellen persönlichen Interessen verbunden sein können.

- Sind oder waren Sie in Wissenschaftlichen Fachgesellschaften, Berufsverbänden, Institutionen der Selbstverwaltung, Patientenselbsthilfegruppen, Verbrauchervertretungen oder anderen Verbänden aktiv? Wenn ja, in welcher Funktion (z.B. Mandatsträger für diese/andere Leitlinien, Vorstand)?
- Können Sie Schwerpunkte Ihrer wissenschaftlichen und /oder klinischen Tätigkeiten benennen? Fühlen Sie sich bestimmten „Schulen" zugehörig?
- Waren Sie an der inhaltlichen Gestaltung von Fortbildungen federführend beteiligt?
- Haben Sie persönliche Beziehungen (als Partner oder Verwandter 1. Grades) zu einem Vertretungsberechtigten eines Unternehmens der Gesundheitswirtschaft?

Machen Sie bitte in folgender Tabelle zu allen zutreffenden Aspekten konkrete Angaben.

Art der Beziehung/Tätigkeit	Namen / Schwerpunkte (bitte konkret benennen)	Zeitraum der Beziehung/ Tätigkeit[7]	Themenbezug zur Leitlinie[8]
Mitgliedschaft /Funktion in Interessenverbänden	Deutsche Gesellschaft für Anästhesiologie und Intensivmedizin e.V. (DGAI, Nürnberg)<br>• Sprecher des Wissenschaftlichen Arbeitskreis Schmerzmedizin, 01/20 bis heute<br>• Standortvertreter Göttingen des Wiss. Arbeitskreis Wissenschaftlicher Nachwuchs, 2019 bis heute<br><br>Deutsche Schmerzgesellschaft e.V. (Berlin)<br>• 2. Sprecher des Arbeitskreises Akutschmerz, 01/2016<br>• Mitglied der wiss. Programmkommission Deutscher Schmerzkongress (2018/2019/2020)<br>• Mitglied Ad-hoc-Kommission „Kongressfortbildung beim Deutschen Schmerzkongress", 2017 bis heute<br>• Mitglied der Ad-hoc-Kommission „Zertifizierung"<br>• Wiss. Leitung „Seeheimer Akutschmerztage", 2016 bis heute<br>• Wiss. Leitung (komm.) „Akutschmerzkurse", 01/2020 bis heute	siehe 1. Spalte	kein direkter Bezug, Vertreter von 2 beteiligten Fachgesellschaften

[7] Innerhalb des Erfassungszeitraums, d.h. im gegenwärtigen und den zurückliegenden 3 Jahren, Angabe: von (Monat/Jahr) bis (Monat/Jahr)
[8] Angabe einer Selbsteinschätzung „Nein" oder „Ja"

	Mitgliedschaften ohne aktive Funktion:<br><br>Deutsche Migräne und Kopfschmerzgesellschaft e.V. (Königstein im Taunus)<br><br>Sertürner Gesellschaft e.V. (Einbeck)<br><br>Göttinger Schmerz- und Palliativverein e.V. (Göttingen)<br><br>European Society of Anaesthesiology (Assoziierte Mitgliedschaft; Brüssel, Belgien)<br><br>International Association for the Study of Pain (IASP, Washington, D.C., USA)		
Schwerpunkte wissenschaftlicher Tätigkeiten, Publikationen	Schmerzchronifizierung und chronischer Schmerz als Komorbidität; Rücken- und Nackenschmerz; Schmerzmedizinische Versorgungsforschung		
Schwerpunkte klinischer Tätigkeiten	Schmerzambulanz, Schmerztagesklinik, Innerklinischer Schmerzdienst		
Federführende Beteiligung an Fortbildungen/Ausbildungsinstituten	siehe oben bei Fachgesellschaften	siehe 1. Spalte	kein direkter Bezug, Vertreter von 2 beteiligten Fachgesellschaften
Persönliche Beziehungen (als Partner oder Verwandter 1. Grades) zu einem Vertretungsberechtigten eines Unternehmens der Gesundheitswirtschaft	keine	-	-

4. Sonstige Interessen

Sehen Sie andere Aspekte oder Umstände, die von Dritten als einschränkend in Bezug auf Ihre Objektivität oder Unabhängigkeit wahrgenommen werden könnten?
nein

Ich erkläre hiermit nach bestem Wissen und Gewissen, dass ich alle mir derzeit bekannten Umstände aufgeführt habe, die gegebenenfalls zu einem persönlichen Interessenkonflikt bei der themenbezogenen Mitwirkung bei der Erstellung der Leitlinie führen können. Ich erkläre weiterhin, dass ich die Diskussion der Erklärungen anderer Mitglieder in der Leitliniengruppe absolut vertraulich behandeln werde. Ich bin darüber informiert, dass die Angaben in standardisierter Zusammenfassung mit der Leitlinie/in einem begleitenden Leitlinienreport veröffentlicht werden, und dass das vorliegende Formular vor der Einsicht unberechtigter Dritter geschützt aufbewahrt wird. Hiermit bin ich einverstanden.

25.6.2020

Datum **Unterschrift**

Ergänzende Hinweise

- Bitte füllen Sie das Formular vollständig aus.
- Falls Sie zu bestimmten Fragen keine Angaben machen können oder wollen, begründen Sie dies bitte.
- Bitte speichern Sie das ausgefüllte Formular und senden es an ulrike.stamer@dbmr.unibe.ch

Erklärung von Interessen

zu Händen

Vorbemerkung

Alle Mitglieder der Leitliniengruppe sind gehalten, die nachstehende Erklärung von Interessen auszufüllen. Die Erklärung wird gegenüber dem Leitlinienkoordinator abgegeben. Dies soll bereits zu Beginn des Leitlinienprojekts erfolgen bzw. zu dem Zeitpunkt, an dem die Mitglieder ihre Teilnahme am Leitlinienprojekt gegenüber dem Koordinator bestätigen. Bei länger andauernden Projekten ist eine Erneuerung der Erklärung einmal pro Jahr bis zum Abschluss der Leitlinienentwicklung, zumindest aber vor der Konsensfindung, erforderlich.

In der Erklärung sind alle Interessen aufzuführen, unabhängig davon, ob der/die Erklärende selbst darin einen thematischen Bezug zur Leitlinie oder einen Interessenskonflikt sieht oder nicht. Ob Interessenkonflikte bestehen und ob dadurch die erforderliche Neutralität für die Mitarbeit bei der Leitlinienentwicklung in Frage gestellt ist oder in welchen speziellen Bereichen /Fragestellungen der Leitlinie das professionelle Urteilsvermögen eines Experten durch sekundäre Interessen beeinflusst sein könnte, ist durch einen Dritten zu bewerten und in der Leitliniengruppe zu diskutieren. Die Erklärung betrifft Interessen innerhalb **des laufenden Jahres sowie der zurückliegenden 3 Jahre.**

Die Originale der Erklärungen verbleiben vertraulich beim Leitlinienkoordinator. Die Inhalte der Erklärungen sind in der Langfassung der Leitlinie bzw. im Leitlinienreport in standardisierter Zusammenfassung offen darzulegen. Ergänzend sind das Verfahren der Sammlung und Bewertung der Erklärungen sowie die Ergebnisse der Diskussion zum Umgang mit Interessenkonflikten darzulegen.

Informationen zur Datenerhebung gemäß Artikel 13 DSGVO

Der Leitlinienkoordinator erhebt Ihre Daten zum Zweck des o.g. Leitlinienvorhabens sowie zur Erfüllung des Regelwerkes der AWMF. Die Datenerhebung und Datenverarbeitung sind für die Durchführung des Leitlinienvorhabens erforderlich und beruhen auf Artikel 6 Abs. 1 b) DSGVO. Eine Weitergabe der Daten an Dritte findet nur zum Zweck der Erfüllung des Regelwerkes der AWMF statt. Die Daten werden gelöscht, sobald sie für den Zweck ihrer Verarbeitung nicht mehr erforderlich sind. Sie sind berechtigt, Auskunft der im Rahmen des Leitlinienvorhabens über Sie gespeicherten Daten zu beantragen sowie bei Unrichtigkeit der Daten die Berichtigung oder bei unzulässiger Datenspeicherung die Löschung der Daten zu fordern.

Erklärung

1. Allgemeine Angaben

Name, Vorname, Titel	Freys, Stephan M., Prof.Dr.med.	
Arbeitgeber / Institution	Gegenwärtig<br>DIAKO Ev. Diakonie-Krankenhaus gemeinnützige Gmbh Bremen	Früher(e) innerhalb des laufenden Jahres oder der 3 Kalenderjahre davor
Position / Funktion in der Institution	Chefarzt Chirurgische Klinik	
Adresse	Gröpelinger Heerstr. 406-408, 28239 Bremen	
e-mail-Adresse	chirurgie@diako-bremen.de	
Bei Rückfragen telefonisch zu erreichen unter	0421 / 6102 1101	
Funktion in der Leitliniengruppe	Mitglied, Mandatsträger DGCH	
Datum	27.02.2020	
Zeitraum, auf den sich die Erklärung bezieht	2017 bis heute	

2. Direkte, finanzielle Interessen

Hier werden finanzielle Beziehungen zu Unternehmen, Institutionen oder Interessenverbänden im Gesundheitswesen erfasst. Haben Sie oder die Einrichtung, für die Sie tätig sind, innerhalb des laufenden Jahres oder der 3 Kalenderjahre davor Zuwendungen erhalten von Unternehmen der Gesundheitswirtschaft (z.B. Arzneimittelindustrie, Medizinproduktindustrie), industriellen Interessenverbänden, kommerziell orientierten Auftragsinstituten, Versicherungen/Versicherungsträgern, oder von öffentlichen Geldgebern (z.B. Ministerien), Körperschaften/Einrichtungen der Selbstverwaltung, Stiftungen, oder anderen Geldgebern? Machen Sie bitte in folgender Tabelle zu allen zutreffenden Aspekten konkrete Angaben.

Art der Beziehung/Tätigkeit	Name des/der Kooperations-partner/s	Zeitraum der Beziehung/ Tätigkeit[1]	Thema, Bezug zur Leitlinie[2]	Art der Zuwendung[3]	Höhe der Zuwendung[4]	Empfänger [5]
Berater-/Gutachtertätigkeit						
Mitarbeit in einem Wissenschaft-lichen Beirat (advisory board)	Grünenthal GmbH	2017 - heute	Orale PCA mit Sufentanil (Zalviso®)	Honorar	7.400,-	DIAKO Ev. Diakonie-Krankenhaus gGmbH Bremen
Vortrags-/oder Schulungstätigkeit						
Autoren-/oder Coautorenschaft						
Forschungsvorhaben/ Durchführung klinischer Studien						
Eigentümerinteressen (Patent, Urheberrecht, Aktienbesitz[6])						

[1] Innerhalb des Erfassungszeitraums, d.h.im gegenwärtigen und den zurückliegenden 3 Jahren, Angabe: von (Monat/Jahr) bis (Monat/Jahr)
[2] Angabe des Themas, bei Präparaten/Geräten auch Handelsname bzw. Wirkstoffname (Freitext), zusätzlich Angabe einer Selbsteinschätzung des Bezugs zur Leitlinie: „Nein" oder „Ja"
[3] Honorar, Drittmittel, geldwerte Vorteile (z.B. Personal-oder Sachmittel; Reisekosten, Teilnahmegebühren, Bewirtung i.R. von Veranstaltungen), Verkaufslizenz
[4] Es können gerundete Beträge angeben werden (z.B. bei Beiträgen > 1000 € jeweils auf die nächste Tausenderstelle): Die Angaben beziehen sich auf die Gesamtsumme der Zuwendungen für eine angegebene Tätigkeit über den Erfassungszeitraum, Angabe: von (Monat/Jahr) bis (Monat/Jahr).
Diese Angaben werden vertraulich behandelt.
[5] Bitte angeben: a) wenn Sie persönlich Empfänger der Zuwendung sind oder b) wenn es die Institution ist, für die Sie tätig sind und Sie innerhalb Ihrer Institution direkt entscheidungsverantwortlich für die Verwendung der Zuwendung/Mittel sind. Sind Sie nicht direkt entscheidungsverantwortlich, sind keine Angaben nötig.
[6] Betrifft nur Eigentümerinteressen im Gesundheitswesen; auch sind Angaben zu Mischfonds nicht erforderlich.

3. Indirekte Interessen

Hier werden persönliche Beziehungen zu Interessenverbänden im Gesundheitswesen, „intellektuelle", akademische, und wissenschaftliche Interessen oder Standpunkte sowie Schwerpunkte klinischer Tätigkeiten/Einkommensquellen erfasst (für den Zeitraum des laufenden Jahres oder der 3 Kalenderjahre davor). Hierunter fallen auch solche, die indirekt mit finanziellen persönlichen Interessen verbunden sein können.

- Sind oder waren Sie in Wissenschaftlichen Fachgesellschaften, Berufsverbänden, Institutionen der Selbstverwaltung, Patientenselbsthilfegruppen, Verbrauchervertretungen oder anderen Verbänden aktiv? Wenn ja, in welcher Funktion (z.B. Mandatsträger für diese/andere Leitlinien, Vorstand)?
- Können Sie Schwerpunkte Ihrer wissenschaftlichen und /oder klinischen Tätigkeiten benennen? Fühlen Sie sich bestimmten „Schulen" zugehörig?
- Waren Sie an der inhaltlichen Gestaltung von Fortbildungen federführend beteiligt?
- Haben Sie persönliche Beziehungen (als Partner oder Verwandter 1. Grades) zu einem Vertretungsberechtigten eines Unternehmens der Gesundheitswirtschaft?

Machen Sie bitte in folgender Tabelle zu allen zutreffenden Aspekten konkrete Angaben.

Art der Beziehung/Tätigkeit	Namen / Schwerpunkte (bitte konkret benennen)	Zeitraum der Beziehung/ Tätigkeit[7]	Themenbezug zur Leitlinie[8]
Mitgliedschaft /Funktion in Interessenverbänden	Vorstandsmitglied Bremer Krebsgesellschaft<br>Vorstandmitglied Arbeitsgemeinschaft Deutscher Darmkrebszentren (addz)<br>Vorstandsmitglied Vereinigung Norddeutscher Chirurgen (NDCH)<br>Vorsitzender Verband Leitender Krankenhausärzte (VLK Bremen)<br>Vorsitzender Chirurgische Arbeitsgemeinschaft Akutschmerz (CAAS) der Deutschen Gesellschaft für Chirurgie (DGCH)	Seit 2014<br>Seit 2011<br>Seit 2013<br>Seit 2018<br>Seit 2013	Kein<br>Kein<br>Kein<br>Kein<br>Kein
Schwerpunkte wissenschaftlicher Tätigkeiten, Publikationen			
Schwerpunkte klinischer Tätigkeiten			
Federführende Beteiligung an Fortbildungen/Ausbildungsinstituten			
Persönliche Beziehungen (als Partner oder Verwandter 1. Grades) zu einem Vertretungsberechtigten eines Unternehmens der Gesundheitswirtschaft			

[7] Innerhalb des Erfassungszeitraums, d.h. im gegenwärtigen und den zurückliegenden 3 Jahren, Angabe: von (Monat/Jahr) bis (Monat/Jahr)
[8] Angabe einer Selbsteinschätzung „Nein" oder „Ja"

4. Sonstige Interessen

Sehen Sie andere Aspekte oder Umstände, die von Dritten als einschränkend in Bezug auf Ihre Objektivität oder Unabhängigkeit wahrgenommen werden könnten?
nein

Ich erkläre hiermit nach bestem Wissen und Gewissen, dass ich alle mir derzeit bekannten Umstände aufgeführt habe, die gegebenenfalls zu einem persönlichen Interessenkonflikt bei der themenbezogenen Mitwirkung bei der Erstellung der Leitlinie führen können. Ich erkläre weiterhin, dass ich die Diskussion der Erklärungen anderer Mitglieder in der Leitliniengruppe absolut vertraulich behandeln werde. Ich bin darüber informiert, dass die Angaben in standardisierter Zusammenfassung mit der Leitlinie/in einem begleitenden Leitlinienreport veröffentlicht werden, und dass das vorliegende Formular vor der Einsicht unberechtigter Dritter geschützt aufbewahrt wird. Hiermit bin ich einverstanden.

27.02.2020
Datum

Unterschrift

Ergänzende Hinweise

- Bitte füllen Sie das Formular vollständig aus.
- Falls Sie zu bestimmten Fragen keine Angaben machen können oder wollen, begründen Sie dies bitte.
- Bitte speichern Sie das ausgefüllte Formular und senden es an das Leitliniensekretariat: xxx@yyy.zz

Erklärung

1. Allgemeine Angaben

Name, Vorname, Titel	Stammschulte, Thomas, Dr. med.	
Arbeitgeber / Institution	Gegenwärtig<br><br>Arzneimittelkommission der deutschen Ärzteschaft (AkdÄ)	Früher(e) innerhalb des laufenden Jahres oder der 3 Kalenderjahre davor<br><br>Ebenfalls AkdÄ
Position / Funktion in der Institution	Referemt Pharmakovigilanz	
Adresse	Herbert-Lewin-Platz 1, 10629 Berlin	
e-mail-Adresse	thomas.stammschulte@akdae.de	
Bei Rückfragen telefonisch zu erreichen unter	030 400445561	
Funktion in der Leitliniengruppe	Coautor	
Datum	9.6.2020	
Zeitraum, auf den sich die Erklärung bezieht	2017 bis 2020	

2. Direkte, finanzielle Interessen

Hier werden finanzielle Beziehungen zu Unternehmen, Institutionen oder Interessenverbänden im Gesundheitswesen erfasst. Haben Sie oder die Einrichtung, für die Sie tätig sind, innerhalb des laufenden Jahres oder der 3 Kalenderjahre davor Zuwendungen erhalten von Unternehmen der Gesundheitswirtschaft (z.B. Arzneimittelindustrie, Medizinproduktindustrie), industriellen Interessenverbänden, kommerziell orientierten Auftragsinstituten, Versicherungen/Versicherungsträgern, oder von öffentlichen Geldgebern (z.B. Ministerien), Körperschaften/Einrichtungen der Selbstverwaltung, Stiftungen, oder anderen Geldgebern? Machen Sie bitte in folgender Tabelle zu allen zutreffenden Aspekten konkrete Angaben.

Art der Beziehung/Tätigkeit	Name des/der Kooperationspartner/s	Zeitraum der Beziehung/ Tätigkeit[1]	Thema, Bezug zur Leitlinie[2]	Art der Zuwendung[3]	Höhe der Zuwendung[4]	Empfänger [5]
Berater-/Gutachtertätigkeit						
Mitarbeit in einem Wissenschaft-lichen Beirat (advisory board)						
Vortrags-/oder Schulungstätigkeit	Vorträge auf Fortbildungsveranstaltungen verschiedener Landessärztekammern				ca. 2000 Euro in den letzten 3 Jahren	
Autoren-/oder Coautorenschaft						
Forschungsvorhaben/ Durchführung klinischer Studien						

[1] Innerhalb des Erfassungszeitraums, d.h.im gegenwärtigen und den zurückliegenden 3 Jahren, Angabe: von (Monat/Jahr) bis (Monat/Jahr)
[2] Angabe des Themas, bei Präparaten/Geräten auch Handelsname bzw. Wirkstoffname (Freitext), zusätzlich Angabe einer Selbsteinschätzung des Bezugs zur Leitlinie: „Nein" oder „Ja"
[3] Honorar, Drittmittel, geldwerte Vorteile (z.B. Personal-oder Sachmittel; Reisekosten, Teilnahmegebühren, Bewirtung i.R. von Veranstaltungen), Verkaufslizenz
[4] Es können gerundete Beträge angeben werden (z.B. bei Beiträgen > 1000 € jeweils auf die nächste Tausenderstelle): Die Angaben beziehen sich auf die Gesamtsumme der Zuwendungen für eine angegebene Tätigkeit über den Erfassungszeitraum, Angabe: von (Monat/Jahr) bis (Monat/Jahr).
Diese Angaben werden vertraulich behandelt.
[5] Bitte angeben: a) wenn Sie persönlich Empfänger der Zuwendung sind oder b) wenn es die Institution ist, für die Sie tätig sind und Sie innerhalb Ihrer Institution direkt entscheidungsverantwortlich für die Verwendung der Zuwendung/Mittel sind. Sind Sie nicht direkt entscheidungsverantwortlich, sind keine Angaben nötig.

Eigentümerinteressen (Patent, Urheberrecht, Aktienbesitz[6])						

[6] Betrifft nur Eigentümerinteressen im Gesundheitswesen; auch sind Angaben zu Mischfonds nicht erforderlich.

3. Indirekte Interessen

Hier werden persönliche Beziehungen zu Interessenverbänden im Gesundheitswesen, „intellektuelle“, akademische, und wissenschaftliche Interessen oder Standpunkte sowie Schwerpunkte klinischer Tätigkeiten/Einkommensquellen erfasst (für den Zeitraum des laufenden Jahres oder der 3 Kalenderjahre davor). Hierunter fallen auch solche, die indirekt mit finanziellen persönlichen Interessen verbunden sein können.

- Sind oder waren Sie in Wissenschaftlichen Fachgesellschaften, Berufsverbänden, Institutionen der Selbstverwaltung, Patientenselbsthilfegruppen, Verbrauchervertretungen oder anderen Verbänden aktiv? Wenn ja, in welcher Funktion (z.B. Mandatsträger für diese/andere Leitlinien, Vorstand)?
- Können Sie Schwerpunkte Ihrer wissenschaftlichen und /oder klinischen Tätigkeiten benennen? Fühlen Sie sich bestimmten „Schulen“ zugehörig?
- Waren Sie an der inhaltlichen Gestaltung von Fortbildungen federführend beteiligt?
- Haben Sie persönliche Beziehungen (als Partner oder Verwandter 1. Grades) zu einem Vertretungsberechtigten eines Unternehmens der Gesundheitswirtschaft?

Machen Sie bitte in folgender Tabelle zu allen zutreffenden Aspekten konkrete Angaben.

Art der Beziehung/Tätigkeit	Namen / Schwerpunkte (bitte konkret benennen)	Zeitraum der Beziehung/ Tätigkeit[7]	Themenbezug zur Leitlinie[8]
Mitgliedschaft /Funktion in Interessenverbänden	Mitgliedschaft International Society of Pharmacovigilance (IsoP), Deutsche Gesellschaft für Innere Medizin	ca. 10 Jahre	nein
Schwerpunkte wissenschaftlicher Tätigkeiten, Publikationen			
Schwerpunkte klinischer Tätigkeiten			
Federführende Beteiligung an Fortbildungen/Ausbildungsinstituten			
Persönliche Beziehungen (als Partner oder Verwandter 1. Grades) zu einem Vertretungsberechtigten eines Unternehmens der Gesundheitswirtschaft			

[7] Innerhalb des Erfassungszeitraums, d.h. im gegenwärtigen und den zurückliegenden 3 Jahren, Angabe: von (Monat/Jahr) bis (Monat/Jahr)
[8] Angabe einer Selbsteinschätzung „Nein“ oder „Ja“

4. Sonstige Interessen

Sehen Sie andere Aspekte oder Umstände, die von Dritten als einschränkend in Bezug auf Ihre Objektivität oder Unabhängigkeit wahrgenommen werden könnten?
Nein

Ich erkläre hiermit nach bestem Wissen und Gewissen, dass ich alle mir derzeit bekannten Umstände aufgeführt habe, die gegebenenfalls zu einem persönlichen Interessenkonflikt bei der themenbezogenen Mitwirkung bei der Erstellung der Leitlinie führen können. Ich erkläre weiterhin, dass ich die Diskussion der Erklärungen anderer Mitglieder in der Leitliniengruppe absolut vertraulich behandeln werde. Ich bin darüber informiert, dass die Angaben in standardisierter Zusammenfassung mit der Leitlinie/in einem begleitenden Leitlinienreport veröffentlicht werden, und dass das vorliegende Formular vor der Einsicht unberechtigter Dritter geschützt aufbewahrt wird. Hiermit bin ich einverstanden.

9.6.2020

Datum **Unterschrift**

Ergänzende Hinweise

- Bitte füllen Sie das Formular vollständig aus.
- Falls Sie zu bestimmten Fragen keine Angaben machen können oder wollen, begründen Sie dies bitte.
- Bitte speichern Sie das ausgefüllte Formular und senden es an ulrike.stamer@dbmr.unibe.ch

Medizinische Hochschule Hannover
Klinische Pharmakologie
Prof. Dr. med. Dirk O. Stichtenoth
Carl-Neuberg-Straße 1 • 30625 Hannover
Telefon (05 11) 5 32-28 21 • Fax -27 50

AWMF

Erklärung von Interessen

(Titel, AWMF-Registernummer)

zu Händen

(Leitlinienkoordinator)

Vorbemerkung

Alle Mitglieder der Leitliniengruppe sind gehalten, die nachstehende Erklärung von Interessen auszufüllen. Die Erklärung wird gegenüber dem Leitlinienkoordinator abgegeben. Dies soll bereits zu Beginn des Leitlinienprojekts erfolgen bzw. zu dem Zeitpunkt, an dem die Mitglieder ihre Teilnahme am Leitlinienprojekt gegenüber dem Koordinator bestätigen. Bei länger andauernden Projekten ist eine Erneuerung der Erklärung einmal pro Jahr bis zum Abschluss der Leitlinienentwicklung, zumindest aber vor der Konsensfindung, erforderlich.

In der Erklärung sind alle Interessen aufzuführen, unabhängig davon, ob der/die Erklärende selbst darin einen thematischen Bezug zur Leitlinie oder einen Interessenskonflikt sieht oder nicht. Ob Interessenkonflikte bestehen und ob dadurch die erforderliche Neutralität für die Mitarbeit bei der Leitlinienentwicklung in Frage gestellt ist oder in welchen speziellen Bereichen /Fragestellungen der Leitlinie das professionelle Urteilsvermögen eines Experten durch sekundäre Interessen beeinflusst sein könnte, ist durch einen Dritten zu bewerten und in der Leitliniengruppe zu diskutieren. Die Erklärung betrifft Interessen innerhalb **des laufenden Jahres sowie der zurückliegenden 3 Jahre.**

Die Originale der Erklärungen verbleiben vertraulich beim Leitlinienkoordinator. Die Inhalte der Erklärungen sind in der Langfassung der Leitlinie bzw. im Leitlinienreport in standardisierter Zusammenfassung offen darzulegen. Ergänzend sind das Verfahren der Sammlung und Bewertung der Erklärungen sowie die Ergebnisse der Diskussion zum Umgang mit Interessenkonflikten darzulegen.

<u>Informationen zur Datenerhebung gemäß Artikel 13 DSGVO</u>

Der Leitlinienkoordinator erhebt Ihre Daten zum Zweck des o.g. Leitlinienvorhabens sowie zur Erfüllung des Regelwerkes der AWMF. Die Datenerhebung und Datenverarbeitung sind für die Durchführung des Leitlinienvorhabens erforderlich und beruhen auf Artikel 6 Abs. 1 b) DSGVO. Eine Weitergabe der Daten an Dritte findet nur zum Zweck der Erfüllung des Regelwerkes der AWMF statt. Die Daten werden gelöscht, sobald sie für den Zweck ihrer Verarbeitung nicht mehr erforderlich sind. Sie sind berechtigt, Auskunft der im Rahmen des Leitlinienvorhabens über Sie gespeicherten Daten zu beantragen sowie bei Unrichtigkeit der Daten die Berichtigung oder bei unzulässiger Datenspeicherung die Löschung der Daten zu fordern.

Erklärung

1. Allgemeine Angaben

Name, Vorname, Titel	Stichtenoth, Dirk O., Prof. Dr. med.	
Arbeitgeber / Institution	Gegenwärtig<br><br>Land Niedersachsen, Medizinische Hochschule Hannover (MHH)	Früher(e) innerhalb des laufenden Jahres oder der 3 Kalenderjahre davor:<br>s. links
Position / Funktion in der Institution	Direktor, Institut für Klinische Pharmakologie der MHH	s. links
Adresse	Carl-Neuberg-Str. 1, 30625 Hannover	
e-mail-Adresse	Stichtenoth.Dirk@MH-Hannover.de	
Bei Rückfragen telefonisch zu erreichen unter	Tel: 0511 532-2820, https://www.mh-hannover.de/klinpharm.html	
Funktion in der Leitliniengruppe	Autor mit Expertise für klinische Pharmakologie	
Datum	03.03.2020	
Zeitraum, auf den sich die Erklärung bezieht	2017-2020	

2. Direkte, finanzielle Interessen

Hier werden finanzielle Beziehungen zu Unternehmen, Institutionen oder Interessenverbänden im Gesundheitswesen erfasst. Haben Sie oder die Einrichtung, für die Sie tätig sind, innerhalb des laufenden Jahres oder der 3 Kalenderjahre davor Zuwendungen erhalten von Unternehmen der Gesundheitswirtschaft (z.B. Arzneimittelindustrie, Medizinproduktindustrie), industriellen Interessenverbänden, kommerziell orientierten Auftragsinstituten, Versicherungen/Versicherungsträgern, oder von öffentlichen Geldgebern (z.B. Ministerien), Körperschaften/Einrichtungen der Selbstverwaltung, Stiftungen, oder anderen Geldgebern? Machen Sie bitte in folgender Tabelle zu allen zutreffenden Aspekten konkrete Angaben.

Art der Beziehung/Tätigkeit	Name des/der Kooperations-partner/s	Zeitraum der Beziehung/ Tätigkeit[1]	Thema, Bezug zur Leitlinie[2]	Art der Zuwendung[3]	Höhe der Zuwendung[4]	Empfänger[5]
Berater-/Gutachtertätigkeit	nein	–	–	–	–	–
Mitarbeit in einem Wissenschaft-lichen Beirat (advisory board)	nein	–	–	–	–	–
Vortrags-/oder Schulungstätigkeit	Rheumatologische Fortbildungsakademie GmbH, Berlin	Januar/2017 bis März/2020<br>Vorträge im Curriculum „Management rheumatologischer Erkrankungen im Alter“	Klinische Pharmakologie bei älteren Rheumapatienten<br>ja	Honorar, Reisekosten	Insgesamt 1795,26 Euro	a)

[1] Innerhalb des Erfassungszeitraums, d.h.im gegenwärtigen und den zurückliegenden 3 Jahren, Angabe: von (Monat/Jahr) bis (Monat/Jahr)
[2] Angabe des Themas, bei Präparaten/Geräten auch Handelsname bzw. Wirkstoffname (Freitext), zusätzlich Angabe einer Selbsteinschätzung des Bezugs zur Leitlinie: „Nein“ oder „Ja“
[3] Honorar, Drittmittel, geldwerte Vorteile (z.B. Personal-oder Sachmittel; Reisekosten, Teilnahmegebühren, Bewirtung i.R. von Veranstaltungen), Verkaufslizenz
[4] Es können gerundete Beträge angeben werden (z.B. bei Beiträgen > 1000 € jeweils auf die nächste Tausenderstelle): Die Angaben beziehen sich auf die Gesamtsumme der Zuwendungen für eine angegebene Tätigkeit über den Erfassungszeitraum, Angabe: von (Monat/Jahr) bis (Monat/Jahr).
Diese Angaben werden vertraulich behandelt.
[5] Bitte angeben: a) wenn Sie persönlich Empfänger der Zuwendung sind oder b) wenn es die Institution ist, für die Sie tätig sind und Sie innerhalb Ihrer Institution direkt entscheidungsverantwortlich für die Verwendung der Zuwendung/Mittel sind. Sind Sie nicht direkt entscheidungsverantwortlich, sind keine Angaben nötig.

	DIAPLAN GmbH, Overath	September/2017<br>Vortrag auf Internisten-Tag Niedersachsen	Nierenfunktion und Medikamente: was ist zu beachten?<br>ja	Honorar, Reisekosten	368,78 Euro	a)
	Landesvereinigung für Gesundheit und Akademie für Sozialmedizin Niedersachsen e. V.	Februar/2018<br>Vortrag auf Fortbildungsveranstaltung „Vermeidung von Polymedikation bei koronarer Herzkrankheit“	Polymedikation<br>nein	Honorar, Reisekosten	333,2 Euro	a)
	Krankenhaus St. Joseph-Stift GmbH, Bremen	Oktober/2018<br>Vortrag auf „3. Bremer Geriatrie-Symposium“	Pharmakologie im Alter<br>ja	Honorar, Reisekosten	1052,95 Euro	a)
Autoren-/oder Coautorenschaft	Georg Thieme Verlag KG, Stuttgart	Januar 2017/März 2017<br>Kapitel im Buch „Schmerzmedizin – 1000 Fragen“	Nichtopioide, Koanalgetika, Adjuvante Therapien, Wechselwirkungen, Schwangerschaft und Stillzeit<br>ja	Honorar	110 Euro	a)
	Deutsche Rheuma-Liga Bundesverband e. V., Bonn	April/2017<br>Beitrag Zeitschrift mobil 4/2017	Methotrexat und Konsum koffeinhaltiger Getränke<br>nein	Honorar	26 Euro	a)
Forschungsvorhaben/ Durchführung klinischer Studien	nein	–	–	–	–	–
Eigentümerinteressen (Patent, Urheberrecht, Aktienbesitz[6])	nein	–	–	–	–	–

[6] Betrifft nur Eigentümerinteressen im Gesundheitswesen; auch sind Angaben zu Mischfonds nicht erforderlich.

3. Indirekte Interessen

Hier werden persönliche Beziehungen zu Interessenverbänden im Gesundheitswesen, „intellektuelle", akademische, und wissenschaftliche Interessen oder Standpunkte sowie Schwerpunkte klinischer Tätigkeiten/Einkommensquellen erfasst (für den Zeitraum des laufenden Jahres oder der 3 Kalenderjahre davor). Hierunter fallen auch solche, die indirekt mit finanziellen persönlichen Interessen verbunden sein können.

- Sind oder waren Sie in Wissenschaftlichen Fachgesellschaften, Berufsverbänden, Institutionen der Selbstverwaltung, Patientenselbsthilfegruppen, Verbrauchervertretungen oder anderen Verbänden aktiv? Wenn ja, in welcher Funktion (z.B. Mandatsträger für diese/andere Leitlinien, Vorstand)?
- Können Sie Schwerpunkte Ihrer wissenschaftlichen und /oder klinischen Tätigkeiten benennen? Fühlen Sie sich bestimmten „Schulen" zugehörig?
- Waren Sie an der inhaltlichen Gestaltung von Fortbildungen federführend beteiligt?
- Haben Sie persönliche Beziehungen (als Partner oder Verwandter 1. Grades) zu einem Vertretungsberechtigten eines Unternehmens der Gesundheitswirtschaft?

Machen Sie bitte in folgender Tabelle zu allen zutreffenden Aspekten konkrete Angaben.

Art der Beziehung/Tätigkeit	Namen / Schwerpunkte (bitte konkret benennen)	Zeitraum der Beziehung/ Tätigkeit[7]	Themenbezug zur Leitlinie[8]
Mitgliedschaft /Funktion in Interessenverbänden	Deutsche Gesellschaft für experimentelle und klinische Pharmakologie und Toxikologie e.V. (DGPT) / Mitglied	1/2017 bis 3/2020	ja
	Deutschen Gesellschaft für Klinische Pharmakologie und Therapie (DGKliPha) / Mitglied	1/2017 bis 3/2020	ja
	Deutsche Gesellschaft für Rheumatologie (DGRh) / Mitglied, Beteiligung an Leitlinienerstellung	1/2017 bis 3/2020	ja
	Deutscher Hochschulverband (DHV) / Mitglied	1/2017 bis 3/2020	nein
	Ärztekammer Niedersachsen / Mitglied, Stellvertretender Vorsitzender der Ethikkommission bei der Ärztekammer Nierdersachsen	1/2017 bis 3/2020	nein
Schwerpunkte wissenschaftlicher Tätigkeiten, Publikationen	Arzneimitteltherapiesicherheit, Klinische Pharmakologie des Prostanoidstoffwechsels, Stickstoffmonoxid-Stoffwechsel bei Entzündungen und dessen pharmakologische Beeinflußbarkeit.	1/2017 bis 3/2020	ja
Schwerpunkte klinischer Tätigkeiten	Fachärztlicher klinisch-pharmakologischer Konsiliardienst, Arzneimittelbeauftragter der MHH und Vorsitz der Arzneimittelkommission. Leitung des Zentrums für Arzneimittelsicherheit.	1/2017 bis 3/2020	ja

[7] Innerhalb des Erfassungszeitraums, d.h. im gegenwärtigen und den zurückliegenden 3 Jahren, Angabe: von (Monat/Jahr) bis (Monat/Jahr)
[8] Angabe einer Selbsteinschätzung „Nein" oder „Ja"

Federführende Beteiligung an Fortbildungen/Ausbildungsinstituten	Fortbildungsveranstaltung Pharmakotherapeutische Besprechung, Institut für Klinische Pharmakologie der MHH<br>Fortbildungsveranstaltung Journal Club: Vorstellung und Diskussion aktueller Publikationen, Institut für Klinische Pharmakologie der MHH<br>Modulares Fortbildungsprogramm für klinische Prüfungen der MHH	1/2017 bis 3/2020<br>1/2017 bis 3/2020<br>1/2017 bis 2/2018	ja<br>ja<br>nein
Persönliche Beziehungen (als Partner oder Verwandter 1. Grades) zu einem Vertretungsberechtigten eines Unternehmens der Gesundheitswirtschaft	nein	–	–

4. Sonstige Interessen

Sehen Sie andere Aspekte oder Umstände, die von Dritten als einschränkend in Bezug auf Ihre Objektivität oder Unabhängigkeit wahrgenommen werden könnten?
Nein

Ich erkläre hiermit nach bestem Wissen und Gewissen, dass ich alle mir derzeit bekannten Umstände aufgeführt habe, die gegebenenfalls zu einem persönlichen Interessenkonflikt bei der themenbezogenen Mitwirkung bei der Erstellung der Leitlinie führen können. Ich erkläre weiterhin, dass ich die Diskussion der Erklärungen anderer Mitglieder in der Leitliniengruppe absolut vertraulich behandeln werde. Ich bin darüber informiert, dass die Angaben in standardisierter Zusammenfassung mit der Leitlinie/in einem begleitenden Leitlinienreport veröffentlicht werden, und dass das vorliegende Formular vor der Einsicht unberechtigter Dritter geschützt aufbewahrt wird. Hiermit bin ich einverstanden.

Hannover, 6. März 2020

Datum

Unterschrift

Ergänzende Hinweise

- Bitte füllen Sie das Formular vollständig aus.
- Falls Sie zu bestimmten Fragen keine Angaben machen können oder wollen, begründen Sie dies bitte.
- Bitte speichern Sie das ausgefüllte Formular und senden es an das Leitliniensekretariat: xxx@yyy.zz

Erklärung

1. Allgemeine Angaben

Name, Vorname, Titel	Priv.-Doz. Dr. Stefan Wirz	
Arbeitgeber / Institution	Gegenwärtig<br>GFO-Kliniken Bonn	Früher(e) innerhalb des laufenden Jahres oder der 3 Kalenderjahre davor
Position / Funktion in der Institution	Chefarzt	
Adresse	Schülgenstr. 15, 5304 Bad Honnef	
e-mail-Adresse	anaesthesie@cura.org; stefan.wirz@gfo-kliniken-bonn.de	
Bei Rückfragen telefonisch zu erreichen unter	02224 772 -1194, -1269, -1285	
Funktion in der Leitliniengruppe	Experte, Mitglied der Deutschen Schmerzgesellschaft	
Datum	24.2.2020	
Zeitraum, auf den sich die Erklärung bezieht	Ab 2017	

2. Direkte, finanzielle Interessen

Hier werden finanzielle Beziehungen zu Unternehmen, Institutionen oder Interessenverbänden im Gesundheitswesen erfasst. Haben Sie oder die Einrichtung, für die Sie tätig sind, innerhalb des laufenden Jahres oder der 3 Kalenderjahre davor Zuwendungen erhalten von Unternehmen der Gesundheitswirtschaft (z.B. Arzneimittelindustrie, Medizinproduktindustrie), industriellen Interessenverbänden, kommerziell orientierten Auftragsinstituten, Versicherungen/Versicherungsträgern, oder von öffentlichen Geldgebern (z.B. Ministerien), Körperschaften/Einrichtungen der Selbstverwaltung, Stiftungen, oder anderen Geldgebern? Machen Sie bitte in folgender Tabelle zu allen zutreffenden Aspekten konkrete Angaben.

Art der Beziehung/Tätigkeit	Name des/der Kooperations-partner/s	Zeitraum der Beziehung/ Tätigkeit[1]	Thema, Bezug zur Leitlinie[2]	Art der Zuwendung[3]	Höhe der Zuwendung[4]	Empfänger [5]
Berater-/Gutachtertätigkeit	nein					
Mitarbeit in einem Wissenschaft-lichen Beirat (advisory board)	Kyowa Kirin<br>Sandoz/Hexal<br>Mundipharma		Kein	Honorar		
Vortrags-/oder Schulungstätigkeit	Kyowa Kirin<br>Sandoz/Hexal<br>Grünenthal		Kein	Honoar		
Autoren-/oder Coautorenschaft	nein					
Forschungsvorhaben/ Durchführung klinischer Studien	nein					

Formatiert: Einzug: Hängend: 0,45 cm

[1] Innerhalb des Erfassungszeitraums, d.h.im gegenwärtigen und den zurückliegenden 3 Jahren, Angabe: von (Monat/Jahr) bis (Monat/Jahr)
[2] Angabe des Themas, bei Präparaten/Geräten auch Handelsname bzw. Wirkstoffname (Freitext), zusätzlich Angabe einer Selbsteinschätzung des Bezugs zur Leitlinie: „Nein“ oder „Ja“
[3] Honorar, Drittmittel, geldwerte Vorteile (z.B. Personal-oder Sachmittel; Reisekosten, Teilnahmegebühren, Bewirtung i.R. von Veranstaltungen), Verkaufslizenz
[4] Es können gerundete Beträge angeben werden (z.B. bei Beiträgen > 1000 € jeweils auf die nächste Tausenderstelle): Die Angaben beziehen sich auf die Gesamtsumme der Zuwendungen für eine angegebene Tätigkeit über den Erfassungszeitraum, Angabe: von (Monat/Jahr) bis (Monat/Jahr).
Diese Angaben werden vertraulich behandelt.
[5] Bitte angeben: a) wenn Sie persönlich Empfänger der Zuwendung sind oder b) wenn es die Institution ist, für die Sie tätig sind und Sie innerhalb Ihrer Institution direkt entscheidungsverantwortlich für die Verwendung der Zuwendung/Mittel sind. Sind Sie nicht direkt entscheidungsverantwortlich, sind keine Angaben nötig.

Eigentümerinteressen (Patent, Urheberrecht, Aktienbesitz[6])	nein					

[6] Betrifft nur Eigentümerinteressen im Gesundheitswesen; auch sind Angaben zu Mischfonds nicht erforderlich.

3. Indirekte Interessen

Hier werden persönliche Beziehungen zu Interessenverbänden im Gesundheitswesen, „intellektuelle“, akademische, und wissenschaftliche Interessen oder Standpunkte sowie Schwerpunkte klinischer Tätigkeiten/Einkommensquellen erfasst (für den Zeitraum des laufenden Jahres oder der 3 Kalenderjahre davor). Hierunter fallen auch solche, die indirekt mit finanziellen persönlichen Interessen verbunden sein können.

- Sind oder waren Sie in Wissenschaftlichen Fachgesellschaften, Berufsverbänden, Institutionen der Selbstverwaltung, Patientenselbsthilfegruppen, Verbrauchervertretungen oder anderen Verbänden aktiv? Wenn ja, in welcher Funktion (z.B. Mandatsträger für diese/andere Leitlinien, Vorstand)?
- Können Sie Schwerpunkte Ihrer wissenschaftlichen und /oder klinischen Tätigkeiten benennen? Fühlen Sie sich bestimmten „Schulen“ zugehörig?
- Waren Sie an der inhaltlichen Gestaltung von Fortbildungen federführend beteiligt?
- Haben Sie persönliche Beziehungen (als Partner oder Verwandter 1. Grades) zu einem Vertretungsberechtigten eines Unternehmens der Gesundheitswirtschaft?

Machen Sie bitte in folgender Tabelle zu allen zutreffenden Aspekten konkrete Angaben.

Art der Beziehung/Tätigkeit	Namen / Schwerpunkte (bitte konkret benennen)	Zeitraum der Beziehung/ Tätigkeit[7]	Themenbezug zur Leitlinie[8]
Mitgliedschaft /Funktion in Interessenverbänden	Deutsche Schmerzgesellschaft, Beirat, Sprcher Arebistkreis Tumorschmerz, Mitlied Ad hoc Kommisssion Konvent der Scherzambulanzen; Mltgleid Arbeitskreis Akutschmerz	seit 2017<br>seit 2007<br>seit 2019<br>seit 2007	kejn
Schwerpunkte wissenschaftlicher Tätigkeiten, Publikationen	Opioidtypische Symptome, Tumorschmerz, Chronifizierung von Schmerzen, Gesichtsschmerz, perioperativer Schmerz	Seit 2000	Kejn
Schwerpunkte klinischer Tätigkeiten	Anästhesie, Schmerzmedizin, Intensivmedizin, Pallitaivmedizin	Seit 1990	Kein
Federführende Beteiligung an Fortbildungen/Ausbildungsinstituten	Unikliniken Bonn<br>GFO-Symposien	2000-2008<br>Seit 2008	Keijn
Persönliche Beziehungen (als Partner oder Verwandter 1. Grades) zu einem Vertretungsberechtigten eines Unternehmens der Gesundheitswirtschaft	nein		

[7] Innerhalb des Erfassungszeitraums, d.h. im gegenwärtigen und den zurückliegenden 3 Jahren, Angabe: von (Monat/Jahr) bis (Monat/Jahr)
[8] Angabe einer Selbsteinschätzung „Nein“ oder „Ja“

4. Sonstige Interessen

Sehen Sie andere Aspekte oder Umstände, die von Dritten als einschränkend in Bezug auf Ihre Objektivität oder Unabhängigkeit wahrgenommen werden könnten?
nein

Ich erkläre hiermit nach bestem Wissen und Gewissen, dass ich alle mir derzeit bekannten Umstände aufgeführt habe, die gegebenenfalls zu einem persönlichen Interessenkonflikt bei der themenbezogenen Mitwirkung bei der Erstellung der Leitlinie führen können. Ich erkläre weiterhin, dass ich die Diskussion der Erklärungen anderer Mitglieder in der Leitliniengruppe absolut vertraulich behandeln werde. Ich bin darüber informiert, dass die Angaben in standardisierter Zusammenfassung mit der Leitlinie/in einem begleitenden Leitlinienreport veröffentlicht werden, und dass das vorliegende Formular vor der Einsicht unberechtigter Dritter geschützt aufbewahrt wird. Hiermit bin ich einverstanden.

26.2. 2020

Datum

Unterschrift

Ergänzende Hinweise

- Bitte füllen Sie das Formular vollständig aus.
- Falls Sie zu bestimmten Fragen keine Angaben machen können oder wollen, begründen Sie dies bitte.
- Bitte speichern Sie das ausgefüllte Formular und senden es an das Leitliniensekretariat: xxx@yyy.zz
